# Association between genes regulating neural pathways for quantitative traits of speech and language disorders

**DOI:** 10.1038/s41525-021-00225-5

**Published:** 2021-07-27

**Authors:** Penelope Benchek, Robert P. Igo, Heather Voss-Hoynes, Yvonne Wren, Gabrielle Miller, Barbara Truitt, Wen Zhang, Michael Osterman, Lisa Freebairn, Jessica Tag, H. Gerry Taylor, E. Ricky Chan, Panos Roussos, Barbara Lewis, Catherine M. Stein, Sudha K. Iyengar

**Affiliations:** 1 Department of Population & Quantitative Health Sciences, Case Western Reserve University, Cleveland, OH USA; 2 Bristol Dental School, Faculty of Health Sciences, University of Bristol, and Bristol Speech and Language Therapy Research Unit, North Bristol NHS Trust, Bristol, UK; 3 Department of Psychological Sciences, Case Western Reserve University, Cleveland, OH USA; 4 Department of Psychiatry, Friedman Brain Institute, and Department of Genetics and Genomic Science and Institute for Multiscale Biology, Icahn School of Medicine at Mount Sinai, New York, NY USA; 5 Department of Pediatrics, Case Western Reserve University, and Rainbow Babies & Children’s Hospital, University Hospital Case Medical Center, Cleveland, OH USA; 6 Nationwide Children’s Hospital Research Institute and Department of Pediatrics, The Ohio State University, Columbus, OH USA; 7 Mental Illness Research, Education, and Clinical Center (VISN 2 South), James J. Peters VA Medical Center, Bronx, NY USA; 8 Cleveland Hearing and Speech Center, Cleveland, OH USA

**Keywords:** Psychiatric disorders, Genome-wide association studies

## Abstract

Speech sound disorders (SSD) manifest as difficulties in phonological memory and awareness, oral motor function, language, vocabulary, reading, and spelling. Families enriched for SSD are rare, and typically display a cluster of deficits. We conducted a genome-wide association study (GWAS) in 435 children from 148 families in the Cleveland Family Speech and Reading study (CFSRS), examining 16 variables representing 6 domains. Replication was conducted using the Avon Longitudinal Study of Parents and Children (ALSPAC). We identified 18 significant loci (combined *p* < 10^−8^) that we pursued bioinformatically. We prioritized 5 novel gene regions with likely functional repercussions on neural pathways, including those which colocalized with differentially methylated regions in our sample. Polygenic risk scores for receptive language, expressive vocabulary, phonological awareness, phonological memory, spelling, and reading decoding associated with increasing clinical severity. In summary, neural-genetic influence on SSD is primarily multigenic and acts on genomic regulatory elements, similar to other neurodevelopmental disorders.

## Introduction

Communication disorders are highly prevalent in the United States with approximately one in twelve children ages 3–17 years demonstrating a disorder^1^. The most common difficulties are a speech problem (5%) or language problem (3.3%). Speech sound disorders (SSD) refer to difficulties producing certain sound past the age that a child is expected to acquire the sound, and include both errors of articulation or phonetic structure (errors due to poor motor abilities associated with the production of speech sounds) and phonological errors (errors in applying linguistic rules to combine sounds to form words). SSD has a prevalence of approximately 16% in children 3 years of age^2^, with an estimated 3.8% of children persisting with speech delay at 6 years of age^3^. More than half of these children encounter later academic difficulties in language, reading, and spelling^4–8^. Because of the clinical heterogeneity of speech problems and their correlation with other communication domains, endophenotypes are key to the study of genetic underpinnings^9^.

Vocabulary is core to speech acquisition^10^. Children with difficulties in speech sound development often have difficulties with oral language and later reading and spelling disability^2,4–6,11^. Thus, speech, language, reading, and spelling measures are highly correlated and often have common genetic associations^7,8^. Moreover, speech and other communication phenotypes follow a developmental trajectory, where some speech and language disorders resolve with age, whereas others persist; genetic influences on the less easily resolved manifestations are generally stronger^12,13^. Because of the common genetic underpinnings and pathologic associations between speech and other communication phenotypes, it is conceivable that genetic replication interweaves with different communication measures. Indeed, various studies have examined candidate gene associations associated with both binary traits and quantitative endophenotypes, and have identified several strong candidates^14^, though a clear model of genetic susceptibility has not emerged. Of seven known GWASs, none overlap in their top results (multiple genes with *p* < 5 × 10^−5^, see Table 2 in the Graham and Fisher meta-analysis paper^13^), because they focused on several phenotypes (word reading, vocabulary, receptive and expressive language, nonword repetition, and language impairment (LI) binary trait), or these measures were assessed at different ages (either pre-school or early school-age)^15–22^. Because these studies only present results from one or a few measures and/or a binary trait, it is difficult to dissect the complexity of shared genetic influences. Most have not focused on children with SSD, particularly measures of articulation. Our sample represents a unique set of deeply phenotyped individuals with information on six domains that form the core of speech and language.

SSDs are likely due to deficits in both motor ability and broader neural dysfunction. While motor deficits contribute to problems in speech production, abnormalities in other neural systems likely influence the formation of phonological representation, which is common to SSD as well as reading and LI. We hypothesize that genomic factors associated with variation in speech production, phonological representation, and language may point to neural pathways common to speech, language, reading, and spelling ability. To address this hypothesis, we examined endophenotypes representing motor speech, vocabulary, phonological memory, phonological awareness, reading, spelling, and language, in order to characterize genetic commonality across these domains and fully characterize the complexity of SSD. We conducted a GWAS in the Cleveland Family Speech and Reading Study (CFSRS), a cohort ascertained through a proband with SSD, and replicated findings in a population-based cohort. We also conducted a methylome-wide study (i.e., MWAS) to determine the functional implications of these genetic associations. We utilized a family-based cohort as our discovery sample because we hypothesized it would be enriched for disease-associated variants^23,24^. In these analyses, we identified new candidate genes for correlated communication endophenotypes, and bioinformatic annotation of these loci revealed that regulation of neural pathways is associated with variation in these measures.

## Results

### Study population

The CFSRS sample included 435 subjects from 148 families (Table 1). Of these, 27% had SSD only, 4% had LI only, 16% had SSD + LI without CAS, and 11% had CAS (Table 1) diagnosed by a speech-language pathologist. There was a high rate of comorbidities, especially among the probands (Table 1, Supplementary Table 4). Of the subjects in the ALSPAC sample, the prevalence of speech problems by parental report varied from 4 to 6% (Supplementary Table 5).

**Table 1:** Characteristic table for CFSRS GWAS sample.

*N*^a^	435
Number of families	148
Median age in children (sd)	6.6 (5.7)
Median age in parents (sd)	36 (8)
Female, *N* (%)	194 (45%)
*Speech disorder subgroup, N (%)*	
CAS	47 (11%)
SSD + LI (no CAS)	70 (16%)
SSD only	119 (27%)
Lang only	17 (4%)
No CAS/SSD/Lang	177 (41%)
Missing	5 (1%)
*Speech disorder subgroup in probands, N (%)*	
CAS	43 (31%)
SSD + LI (no CAS)	39 (28%)
SSD only	49 (36%)
Lang only	3 (2%)
No CAS/SSD/Lang	3 (2%)
*Hollingshead SES*	
1 (lowest)	3 (1%)
2	30 (7%)
3	67 (15%)
4	167 (38%)
5 (highest)	147 (34%)
Missing	21 (5%)

^a^Sample considered is the union of all samples across the 16 tests. Specific test sample sizes and age ranges are shown in Supplementary Table 1.

### Genetic correlation analysis reveals new relationships among endophenotypes

**Fig. 1:**
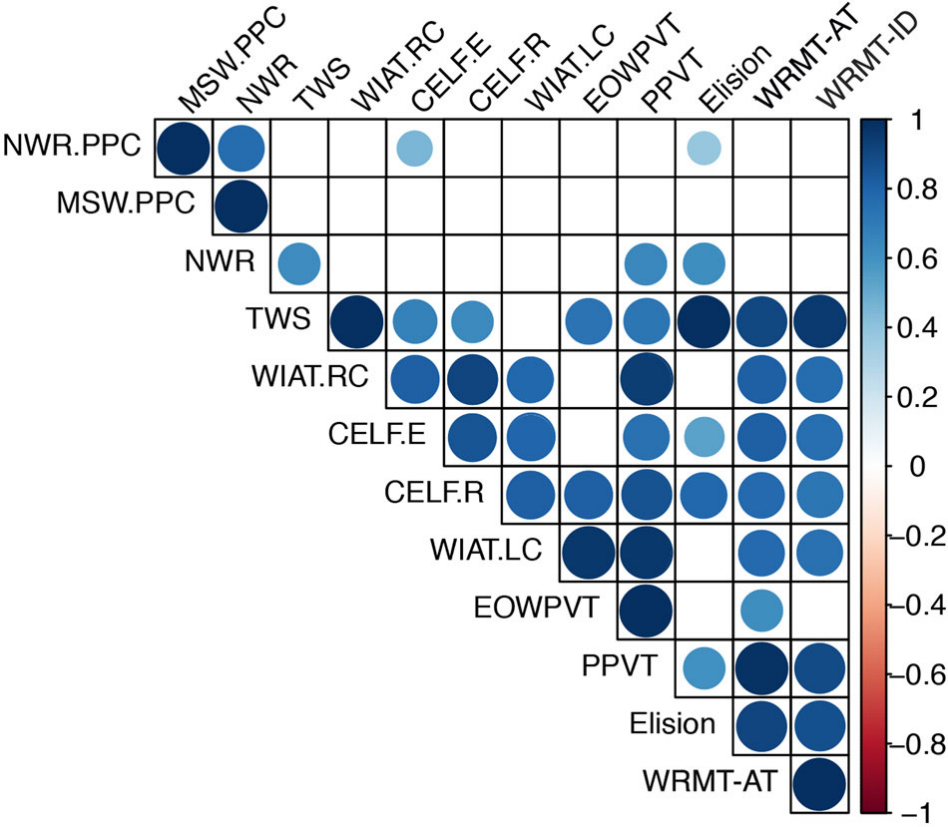
Genetic correlation matrix across traits in CFSRS. Figure 1 shows cross-trait correlation results for each pair of tests using GCTA’s bivariate REML analysis^69^. The cross-trait correlation was tested under the null hypothesis of 0 correlation. Circles shown are for results significant at *p* < 0.05, with increasing diameter/color corresponding with increasing correlation (circles omitted otherwise). Traits: Phonological memory MSW.PPC, MSW, NSW, NSW.PPC), Spelling (TWS), Reading (WRMT-ID, WRMT-AT, WIAT-RC), Language (CELF.E, CELF.R, WIAT.LC), Vocabulary (EOWPVT, PPVT), phonological awareness (Elision).

Genetic correlation analysis revealed that while many of the patterns of correlation were consistent with phenotypic correlations we have previously reported^8^, polygenic correlations enable a deeper understanding of these measures, which will inform the examination of replication of association effects both within the CFSRS data set and with measures from ALSPAC (Fig. 1). For example, while previous studies have demonstrated a strong genetic correlation between reading and spelling measures, polygenic correlation analysis additionally reveals correlations between those skills and Elision. Not surprisingly, expressive and receptive language (measured by the CELF), are strongly correlated with vocabulary (EOWPVT and PPVT) in addition to reading (WRMT-AT and WRMT-ID). Vocabulary is also strongly correlated with listening comprehension (WIAT-LC).

### The most significant findings from GWAS reveal five new candidate genes

Single marker association tests significant at *p* < 10^−5^ were examined further and integrated with data on gene expression and regulation, as detailed below. Other GWAS of neuropsychiatric disease and behavioral traits have similarly found that noncoding regions harbor a significant proportion of risk alleles^25^ (Supplementary Fig. 1).

**Fig. 2:**
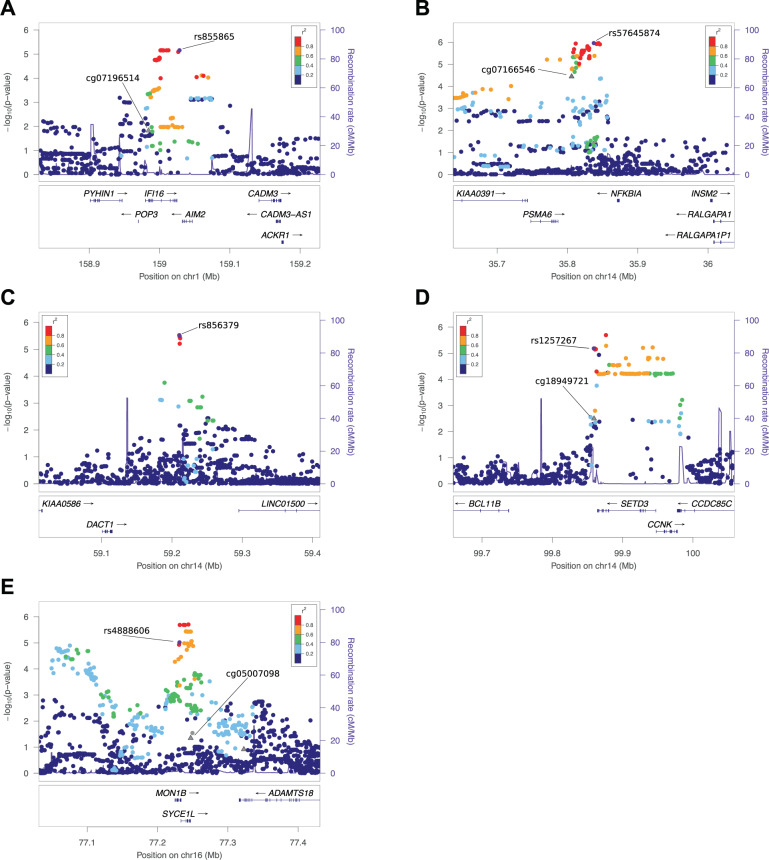
Locus zoom plots for most significant findings. Figure 2 shows association results for the top loci. *p*-Values displayed are for CFSRS and are for the test for which the top SNP was observed. Circles show *p*-values for SNP associations and triangles show *p*-values for methylation associations (specifically those for which the top SNP is an meQTL). The plot shows the top SNP for each region ±200 kb. **a** *IFI16* region. rs855865 was associated with NSW in CFSRS (*p* = 7 × 10^−6^) and with vocabulary (WISC-V) in ALSPAC (*p* = 0.01). This region also includes an meQTL (rs12124059, *p* = 4 × 10^−8^) for methylation marker cg07196514, and this methylation marker (cg07196514) was also associated with NSW (*p* = 0.018). **b** *NFKBIA* region. rs57645874 was associated with Elision in CFSRS (*p* = 1 × 10^−6^) and with reading accuracy (NARA-A) in ALSPAC (*p* = 0.02). This region also contains an meQTL, rs4981288, for cg07166546 (*p* = 2 × 10^−50^), and this methylation marker was associated with Elision (*p* = 3 × 10^−5^), TWS (*p* = 0.0005) and WRMT-ID (*p* = 0.002). **c** *DACT1* region. rs856379 was associated with MSW in CFSRS (*p* = 3 × 10^−6^) and with nonword reading (ALSPACread) in ALSPAC (*p* = 0.036). This SNP is an meQTL for methylation marker cg13972423 (p = 3 × 10^−5^). **d** *SETD3* region rs1257267 was associated with WRMT-AT in CFSRS (*p* = 6.58 × 10^−6^) and with nonsense word repetition (CNrep5) in ALSPAC (*p* = 0.05). While only 1 SNP replicated between CFSRS and ALSPAC, 14 additional SNPs showed association in CFSRS at *p* < 10^−5^. This SNP (rs1257267) is an meQTL for cg18949721 (*p* = 4 × 10^−12^), and methylation marker cg18949721 was also associated with WRMT-AT (*p* = 0.003). **e** *MON1B* region. rs4888606 was associated with MSW in CFSRS (*p* = 9 × 10^−6^) and with nonword reading (ALSPACread) in ALSPAC (*p* = 0.046). While only 1 SNP replicated between CFSRS and ALSPAC, 18 additional SNPs showed association in CFSRS at *p* < 10^−5^. This SNP (rs4888606) falls in an intron of *MON1B* and is an meQTL for cg06128999 (*p* = 4 × 10^−23^) and cg05007098 (*p* = 1 × 10^−15^); these 2 methylation markers were also associated with MSW (*p* = 0.045 and *p* = 0.12, respectively). Functional annotation is in Supplementary Fig. 2.

Of five top loci, all had enhancers or promoters for muscle, brain, and/or neuronal progenitor cells, four out of five had significant methylation and meQTL effects, and three out of five had eQTLs for brain and/or skeletal-muscle tissue (Table 2, Fig. 2, Supplementary Fig. 2, Supplementary Table 6). EpiXcan analysis suggested that the SNP in the chromosome 1 *IFI6* region is associated with expression in the DLPF cortex (Elision *p* = 0.018, TWS *p* = 0.008; Supplementary Tables 7 and 8). The first region on chromosome 14, including *NFKBIA* and *PPP2R3C*, shows significant chromatin interaction mapping in adult cortex tissue. *NFKBIA*, which codes for a component of the NF-κB pathway, is associated with neurogenesis, neuritogenesis, synaptic plasticity, learning, and memory^26^. The second region on chromosome 14 includes *PP2R3C*, which is within the topologically associating domain (TAD) boundary of the *NFKBIA* locus in the Hippocampus and DLPFC. EpiXcan analysis showed *NFKBIZ*, a gene in the same pathway as *NFKIBA*, is also associated with expression in the DLPFC (Elision *p* = 0.000452, TWS *p* = 0.004939; Supplementary Tables 7 and 8). Further, there was significant colocalization at the *MON1B/SYCE1L* locus on chromosome 16, with differential gene expression of *SYCE1L* in multiple brain tissues and skeletal muscle localizing with our SNP association signature (Supplementary Fig. 3) and borderline significant colocalization with *MON1B* expression. The *SETD3* locus also showed colocalization with gene expression in skeletal muscle and brain tissue (Supplementary Fig. 3).

**Table 2:** Annotation of most significant loci with replication in CFSRS and ALSPAC.

Locus (Chr location)	Gene(s)	Associated SNPs^a^	Independently associated SNPs (after conditional analysis)^a^	CFRS Lead SNP MAF	CFRS Lead SNP *p*-value	ALSPAC best replication *p*-value	Fisher combined *p*-value	Locus priority score	Expression in brain/neural tissue	Associated with Communication and/or psych phenotype	Associated with multiple CFSRS traits	Promoter (muscle, brain, neural)	Enhancer (muscle, brain, neural)	eQTL (muscle, brain,neural)	Target of FOXP2 (brain)	Methylation / meQTL
1:30732871	LINC01648;MATN1	1	1	0.14	6.1 × 10^−6^	4.1 × 10^−2^	4.1 × 10^−6^	**3**	1	1	0	1	0	0	0	0
1:55494735*	BSND;PCSK9	5	1	0.42	1.6 × 10^−2^	3.8 × 10^−8^	1.4 × 10^−8^	**4**	1	0	1	1	0	0	0	1
1:146988760	LINC00624	1	1	0.81	6.7 × 10^−6^	4.1 × 10^−3^	5.1 × 10^−7^	**5**	1	1	0	0	1	1	0	1
1:159028378	IFI16, AIM2	23	1	0.27	6.9 × 10^−6^	9.8 × 10^−3^	1.2 × 10^−6^	**5**	0	1	0	0	1	1	0	2
2:143378805	LRP1B;KYNU	4	1	0.76	5.0 × 10^−6^	8.9 × 10^−3^	8.0 × 10^−7^	**4**	1	1	1	1	0	0	0	0
2:169280713	STK39;CERS6	1	1	0.5	2.4 × 10^−6^	4.8 × 10^−2^	2.0 × 10^−6^	**3**	1	1	0	0	1	0	0	0
3:1942898	CNTN6;CNTN4	1	1	0.15	6.3 × 10^−6^	1.8 × 10^−2^	1.9 × 10^−6^	**3**	1	1	0	0	1	0	0	0
3:39743136	MOBP;MYRIP	1	1	0.13	8.9 × 10^−6^	4.5 × 10^−2^	6.3 × 10^−6^	**2**	0	1	0	0	0	0	0	1
4:27297733	LINC02261;MIR4275	9	1	0.09	6.1 × 10^−7^	3.4 × 10^−2^	3.6 × 10^−7^	**2**	1	0	0	0	1	0	0	0
4:73572756	ADAMTS3;COX18	7	1	0.05	2.6 × 10^−6^	4.4 × 10^−2^	2.6 × 10^−6^	**4**	1	1	1	0	1	0	0	0
4:77531588	SHROOM3	1	1	0.14	8.1 × 10^−6^	4.3 × 10^−3^	6.4 × 10^−7^	**3**	1	0	0	1	1	0	0	0
5:72144005	TNPO1	1	1	0.43	2.1 × 10^−6^	3.6 × 10^−2^	1.3 × 10^−6^	**4**	1	1	0	1	1	0	0	0
5:132043351	KIF3A	1	1	0.48	4.0 × 10^−6^	4.7 × 10^−3^	3.6 × 10^−7^	**4**	1	1	0	0	1	1	0	0
5:170102906	KCNIP1	2	1	0.12	5.7 × 10^−6^	3.5 × 10^−2^	3.4 × 10^−6^	**1**	0	0	0	0	0	0	0	1
5:172924967	MIR8056;LOC285593	15	1	0.08	5.6 × 10^−7^	5.8 × 10^−3^	6.7 × 10^−8^	**4**	1	0	0	1	1	0	0	1
7:123604182	SPAM1	10	1	0.13	7.4 × 10^−6^	1.6 × 10^−2^	2.3 × 10^−6^	**4**	1	1	0	1	1	0	0	0
7:154706515	DPP6;PAXIP1-AS2	1	1	0.05	2.5 × 10^−4^	7.2 × 10^−6^	3.9 × 10^−8^	**5**	1	1	0	1	1	0	1	0
9:114335864	PTGR1;ZNF483	0	1	0.24	5.4 × 10^−4^	5.5 × 10^−6^	6.2 × 10^−8^	**6**	1	1	0	1	1	1	0	1
10:46027420	MARCH8	2	1	0.39	1.6 × 10^−6^	3.1 × 10^−2^	9.1 × 10^−7^	**4**	1	1	0	0	1	1	0	0
12:21002703	SLCO1B3	2	1	0.43	6.4 × 10^−6^	7.5 × 10^−3^	8.8 × 10^−7^	**1**	0	0	0	0	0	0	0	1
12:103677691**	LOC101929058; C12orf42	1	1	0.07	1.2 × 10^−5^	5.8 × 10^−5^	1.6 × 10^−8^	**1**	0	1	0	0	0	0	0	0
12:131389783	RAN; ADGRD1	1	1	0.33	7.2 × 10^−6^	4.4 × 10^−3^	5.8 × 10^−7^	**0**	0	0	0	0	0	0	0	0
13:28329109	POLR1D; GSX1	18	1	0.38	7.8 × 10^−6^	3.8 × 10^−2^	4.8 × 10^−6^	**1**	0	0	0	1	0	0	0	0
13:79839523	LINC00331; RBM26	10	1	0.41	1.2 × 10^−6^	4.0 × 10^−2^	8.9 × 10^−7^	**2**	0	1	0	0	0	1	0	0
14:35837476	PSMA6; NFKBIA	26	1	0.19	1.1 × 10^−6^	2.1 × 10^−2^	4.4 × 10^−7^	**7**	1	1	0	1	1	1	0	2
14:59210646	DACT1; LINC01500	7	1	0.05	3.1 × 10^−6^	3.6 × 10^−2^	1.9 × 10^−6^	**5**	1	1	0	1	1	0	0	1
14:93195374	LGMN	1	1	0.14	8.1 × 10^−6^	4.4 × 10^−2^	5.7 × 10^−6^	**4**	1	1	0	1	1	0	0	0
14:94993936*	SERPINA12; SERPINA4	5	1	0.17	1.7 × 10^−1^	3.0 × 10^−9^	5.0 × 10^−8^	**5**	0	1	1	1	1	0	0	1
14:99858970	BCL11B; SETD3	1	1	0.38	6.5 × 10^−6^	4.9 × 10^−2^	5.1 × 10^−6^	**8**	1	1	0	1	1	1	1	2
16:77231207	MON1B	1	1	0.42	9.4 × 10^−6^	4.6 × 10^−3^	6.8 × 10^−6^	**7**	1	1	0	1	1	1	0	2
18:4023876	DLGAP1	1	1	0.19	2.9 × 10^−6^	3.9 × 10^−2^	1.9 × 10^−6^	**3**	1	1	0	0	1	0	0	0
18:40822793	RIT2; SYT4	1	1	0.05	6.8 × 10^−6^	2.8 × 10^−2^	3.2 × 10^−6^	**1**	0	1	0	0	0	0	0	0
18:56462735	MALT1; LINC01926	1	1	0.14	8.8 × 10^−6^	1.1 × 10^−2^	1.5 × 10^−6^	**3**	0	1	0	0	1	0	0	1

^a^Associated SNPs include those associated in CFSRS (*p* < 10^−5^) and Alspac (*p* < 0.05) or Fisher combined *p* < 10^−7^

*Alspac led locus. No CFSRS SNPs showed association at *p* < 10^−5^.

**CFSRS *p* = 1.3 × 10^−5^ and ALSPAC *p* = 5.8 × 10^−5^ (Fisher *p* = 1.6 × 10^−8^).

DACT1 is associated with multiple CFS traits, but not in SNPs that replicated.

*Note* Bold values represents the sum of the column to the right.

### Replication of previous communication disorder loci

In the replication phase, we focused on gene-level replication because of the differences in SNP coverage between our study and the original findings. *ATP2C2* was associated with single word reading (WRMT-ID, *p* = 7.6 × 10^−8^), nonword reading (WRMT-AT, *p* = 4.6 × 10^−5^), and phonological awareness (Elision, *p* = 4.6 × 10^−5^), consistent with prior literature^27^ (Supplementary Figs. 4 and 5). Similarly, *CYP19A1* was associated with nonword reading (WRMT-AT, *p* = 2.8 × 10^−5^), phonological awareness (*p* = 3.3 × 10^−4^), and single-word reading (WRMT-ID, *p* = 5.0 × 10^−4^), validating a previous association^28^. *CNTNAP2* was associated with receptive language (CELF-R, *p* = 5.2 × 10^−6^), and diadochokinetic rate (DDK, *p* = 2.9 × 10^−5^), replicating a previous association^27^. While SNPs within *ROBO1* and *ROBO2* were not significantly associated with our measures, SNPs in the intergenic region were associated with single word reading (WRMT-ID, *p* = 3.6 × 10^−6^); *ROBO1* was originally associated with dyslexia while *ROBO2* was originally associated with expressive vocabulary^22,29^. Finally, SNPs within the *DCDC2-KIAA0319-TTRAP* and in *FOXP2* regions were associated with various traits at *p* < 0.01. Within the ALSPAC cohort, a different pattern of replication emerged (Supplementary Fig. 6), with sometimes different SNPs and/or different phenotypes than those associated with CFSRS.

In addition, we examined loci (genes and/or SNPs) associated in recently published GWAS studies of language and reading^15–22^ (Supplementary Data 2 and 3); we restricted our examination to the CFSRS data, since the ALSPAC data were included in some of the original studies. In these analyses, we often observed cross-trait replication, with most genes originally associated with dyslexia, and associated with other traits in our sample. These included *ZNF385D*^16^, which was associated with all CFSRS traits at *p* < 0.005, *CDH13*^21^, associated with all CFSRS traits at *p* < 0.005, *GRIN2B*^17^, associated with spelling (TWS), expressive vocabulary (EOWPVT), and phonological awareness (Elision) at *p* < 0.0005 and all CFSRS traits at *p* < 0.05, *NKAIN*^17^, associated with receptive language (CELF-R, at 9.7 × 10^−5^ (rs16928927 *p* = 1 × 10^−4^) and reading comprehension (WIAT-RC, *p* = 4 × 10^−4^), and *MACROD2*^19^ associated with all CFSRS traits at *p* < 0.005).

### Polygenic risk scores are associated with increasing clinical severity

**Fig. 3:**
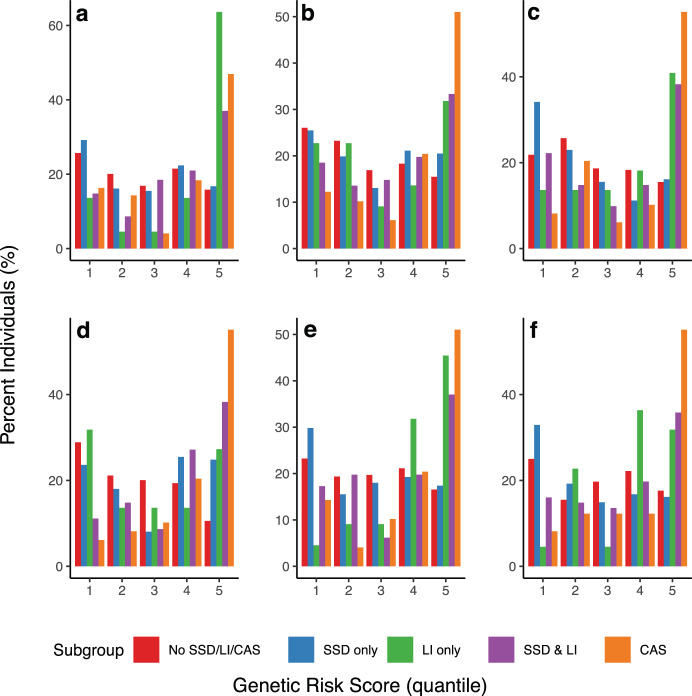
Polygenic risk scores across major domains. We constructed polygenic risk scores for 587 individuals who were both genotyped and had clinical subgroup information available. Polygenic risk scores are displayed by quantile across the clinical subgroups for six endophenotypes representing the major domains (**a** Receptive language; **b** Expressive vocabulary; **c** Phonological awareness; **d** Phonological memory; **e** Spelling; **f** Reading decoding).

In Fig. 3, we illustrate polygenic risk scores (PRS) for six endophenotypes representing the major domains that we analyzed (receptive language, expressive vocabulary, phonological awareness, phonological memory, spelling, and reading decoding), by quintile, across the clinical subgroups to illustrate the connection between clinical diagnosis and genetic underpinnings of these traits (all endophenotypes are illustrated in Supplementary Fig. 7). Generally, we found that polygenic load, indicated by increasing risk scores, was associated with clinical severity (*p* < 1 × 10^−8^ by ANOVA), with typical children having the lowest scores, followed by children with SSD-only, and children with SSD + LI and CAS having the greatest scores. The exception to this trend is receptive language, where the genetic load is greatest for children with LI, for whom receptive language is a focal deficit. Thus, in general, an increase in PRS scores is associated with greater clinical severity.

## Discussion

Communication disorders are genetically complex, manifested by a variety of deficiencies in articulation, vocabulary, receptive and expressive language, phonological awareness, reading decoding and comprehension, and spelling. This study ascertained children through an earlier-presenting clinical disorder and examined several key communication measures, and is thus one of the first studies of its kind. This study is also novel in that it is the first GWAS to include a measure of phonological awareness, as well as a motor speech measure. By analyzing several endophenotypes together, we can draw conclusions about the common genetic basis across these seemingly dissimilar skills. Here, we have identified five new candidate regions, some containing multiple genes, that have connections to neurological function and regulation of neurological pathways. We also found that increased polygenic load is associated with more severe communication disorders. Finally, by examining genetic correlations among these traits, we conclude that different domains of communication have some common genetic influences. All of these aspects together add new clarity regarding the genetic underpinnings of speech and language skills.

First, the novel candidate genes that we have identified all have roles in neurological function as evidenced by expression levels of those genes in brain and/or neural tissue, and associations with other communication and/or psychiatric phenotypes. Colocalization analysis provided the strongest evidence for two loci, *MON1B/SYCE1L* and *SETD3*, showing that our association effects localized with gene expression in brain and skeletal tissue. This commonality between communication traits and brain and neural pathways was also demonstrated by a mouse study of vocalization^30^, and pleiotropy between the brain, learning, and psychiatric phenotypes was recently demonstrated by a large GWAS of brain phenotypes^31^. The existence of enhancers, promoters, and methylation effects in the associated regions further emphasizes the importance of regulatory effects on these traits. Deletions spanning *SETD3* and *CCNK* have been associated with syndromic neurodevelopmental disorders^32^ and variants in *SETX*, within this same family of genes, have been associated with CAS^33^. In addition, *CCNK* is in the *FOXP2* pathway in brain tissue^34–36^. *NFKBIA* is involved in the regulation of the NF-κB pathway, which is involved a number of brain-related processes including neurogenesis, neuritogenesis, synaptic plasticity, learning, and memory^37^. *PPP2R3C* has been associated with schizophrenia^38^. *IFI6* expression has been associated with autism^39^ and overexpression of *IFI6* in the brain is present in chronic neurodegeneration^40^. Finally, *DACT1* may be involved in excitatory synapse organization and dendrite formation during neuronal differentiation^41^ and is mainly expressed within the first two trimesters of pregnancy, just before the first evidence of speech processing is observed in preterm neonates^42^. *DACT1* was associated with several endophenotypes in our sample. Interestingly, *SETD3, NFKBIA*, and *IFI6* are all also tied to the immune system, and a recent study identified an excess of T cells in the brains of individuals with autism^43^.

Second, understanding the genetic architecture across these endophenotypes is essential for understanding how loci are associated with different measures in different study cohorts or across the developmental trajectory. Strong genetic correlations are observed between spelling, reading comprehension and decoding, expressive and receptive language, vocabulary, and phonological awareness. The strongest replications were for a variety of measures collected in CFSRS with ALSPAC from older youth. Consistent with these findings, we previously demonstrated that spelling at later ages has a higher estimated heritability than spelling at school-age^12^. Measures administered in older youth may also be more sensitive to variations in clinical manifestation of SSD. Examination of the ALSPAC measures suggests that many of those administered at younger ages may have tapped different domains than intended, or may have been less sensitive to later emerging reading and spelling skills. Methods of cohort ascertainment may also be important in comparing our findings to those of other studies. Our families were ascertained through a child with SSD whereas other studies ascertained subjects through LI or dyslexia. These different ascertainment schemes affect both the available measures, as well as the distribution of scores and power to detect association. Since dyslexia emerges later than SSD, longitudinal studies that ascertain through a proband with SSD will be able to capture variants associated with SSD, LI, and dyslexia, as there is high comorbidity. In addition to the plethora of studies ascertaining children at a variety of ages, which has an impact on the heritability of traits^7^, these studies use a wide variety of measures, even for the same endophenotype. Moreover, these studies have been conducted in populations that speak different languages of varying orthographic transparency, which makes them difficult to compare. As noted by Carrion-Castillo et al.^15^, most of the novel loci identified through GWAS have been unique to each study, and these aforementioned issues may explain the lack of replication. Thus, examination of the genetic correlation matrix is essential for the interpretation of results across studies, as it is nearly impossible to analyze the same exact traits, as we have demonstrated with our replication study cohort (ALSPAC).

Third, we replicated candidate genes that had been previously primarily associated with reading and/or LI: *CNTNAP2, ATP2C2*, and *CYP19A1*. These analyses extend previous findings to show that these genes are associated with articulation (*CNTNAP2*) and phonological awareness (*ATP2C2* and *CYP19A1*). This further illustrates the pleiotropic nature of these genes. While we did not observe an association with SNPs within the coding regions of *ROBO1* and *ROBO2*, we did observe significant associations with SNPs between these two genes, which may have regulatory influences on *ROBO1/ROBO2*. We also replicated (*p* < 5 × 10^−3^) loci identified in recent GWAS of reading and/or language traits. Similar to another association study between *FOXP2* variants and language^44^, we did not observe a statistically significant association between *FOXP2* and measures in CFSRS, though there was a replication of some traits at a less stringent (*p* < 0.01) level^44^.

Finally, our analysis of PRSs shows strong associations between these risk scores and clinical outcomes of increasing severity. Because of the strong significance of these findings, this suggests that the genetic architecture of communication disorders are maybe largely polygenic, which may additionally explain the lack of replication and/or genome-wide significance. While other studies have examined PRSs associated with language^17,45^, ours is the first to examine the polygenic risk associated with other communication endophenotypes. It is noteworthy that our associated SNPs fell outside of gene coding regions but resided in regulatory regions, even having potential regulatory effects themselves as further evidenced by colocalization analysis. This further illustrates the genetic complexity of communication disorders; perhaps the search for single gene dysfunction is misplaced, and rather regulatory functions are more relevant.

This study has several limitations. The sample size of the CFSRS cohort was modest, potentially reducing power. There was no clear correspondence between measures obtained in ALSPAC with those in CFSRS, necessitating consideration of cross-trait replication. We restricted analyses in both cohorts to individuals of European descent because of the low sample size in other ethnic groups, reducing generalizability.

In summary, this first GWAS of communication measures ascertained through families with SSD identified five new candidate genes, all with potential relevance in central nervous system function. Polygenic risk is strongly associated with more severe speech and language outcomes. Careful consideration of genetic correlation among domains of verbal and written language shows that these loci have general effects on communication, not specific to any single domain, suggesting a common genetic architecture. Further research is needed to more closely examine the impact of regulatory variants on these outcomes.

## Methods

### Subject ascertainment—CFSRS

From the CFSRS^46–51^, we examined 435 individuals from 148 families who had both DNA and endophenotype data available (Table 1). As previously described, families were ascertained through a proband with SSD identified from caseloads of speech-language pathologists in the Greater Cleveland area and referred to the study. All participants met inclusion criteria based on information provided by a parent in an interview or via questionnaire including normal hearing acuity; fewer than six episodes of otitis media prior to age 3; monolingual English speaker; absence of a history of neurological disorders other than childhood apraxia of speech (CAS), such as cerebral palsy or autism spectrum disorder; and a diagnosis of an SSD or suspected CAS by a local speech-language pathologist or neurologist. Diagnosis of CAS, one severe type of SSD, was confirmed by an experienced licensed speech-language pathologist upon enrollment into the study. Socioeconomic status was determined at the initial assessment based on parent education levels and occupations using the Hollingshead Four Factor Index of Social Class^52^. This study was approved by the Institutional Review Board of Case Medical Center and University Hospitals and all parents provided informed consent and children older than 5 years provided assent.

### Communication measures in CFSRS

We studied many endophenotypes covering domains that are common to speech, language, and reading, We examined diadochokinetic rates using the *Robbins and Klee Oral Speech Motor Control Protocol*^53^ or *Fletcher Time-by-Count Test of Diadochokinetic Syllable Rate*^54^. The merged variable is referred to as DDK. Expressive vocabulary was assessed with the *Expressive One Word Picture Vocabulary Test-Revised* (*EOWPVT*^55^*)* and receptive vocabulary with the *Peabody Picture Vocabulary Test—Third Edition (PPVT*^56^*)*, and phonological memory with the *Nonsense Word Repetition (NSW*^57^*), Multisyllabic Word Repetition (MSW*^57^*), and Rapid Color Naming*^58^ task. In addition to examining the total number of words correct for the MSW and NSW, we also examined the percent phonemes correct for both of these tasks (NSW-PPC and MSW-PPC, respectively). Phonological awareness was assessed using the *Elision* subtest of the *Comprehensive Test of Phonological Processing*—Second Edition^59^, which measures the ability to remove phonological segments from spoken words to form other words. Reading was assessed using the *Woodcock Reading Mastery Test-Revised, Word Attack subtest (WRMT-AT*) (reading of nonsense words) and *Word Identification Subtest (WRMT-ID)* (reading of real words), the *Reading Comprehension subtest (WIAT-RC)*, and *Listening Comprehension subtest (WIAT-LC)* of the *Wechsler Individual Achievement Test*^60^. Spelling was assessed on the *Test of Written Spelling-3 (TWS)* using the total score^61^. The expressive and receptive language was assessed using the *Test of Language Development (TOLD*^62^*)* and *Clinical Evaluation of Language Fundamentals-Revised* or *Clinical Evaluation of Language Fundamentals-Preschool* according to age *(CELF*^63^*)* referred to as the CELF-E (expressive) and CELF-R (receptive), respectively. Additional details about these measures are provided in the Supplementary Note. For each of our tests, we selected the first available assessment for each individual (Supplementary Table 1).

For the following tests—NSW, NSW PPC, MSW, and MSW PPC—we did not have population normed data, therefore, we converted all scores to age-adjusted *z*-scores using CFSRS controls. Here, controls were defined as individuals without SSD, LI or CAS. To age-adjust we chose the first available observation for each of the four tests for every control within the CFSRS to determine the effect of age. The age-adjusted score is simply the standardized residual of the score with the effect of age and age-squared regressed out (where the age effect is determined by controls and subsequent adjustment is applied to all participants)^48,64^. Age and age-squared are both used to determine the effect of age, as there is a nonlinear relationship between age and each of the above four tests. If applicable, test scores were transformed to an approximately normal distribution using the Box–Cox power transformation^39^. Because measures were already age-normed or age-adjusted, age was not included additionally as a covariate in GWAS or other analytical models.

### GWAS analysis

DNA was extracted from buffy coats or saliva samples as previously described^6^. All genotyping was performed using the Illumina Omni 2.5 platform. Standard QC procedures were applied, including filtering based on call rate, Hardy–Weinberg equilibrium (HWE), chromosome (autosomes only), minor allele frequency (MAF), and Mendelian errors. Principal components analysis (PCA) was conducted using markers that attained MAF ≥ 0.01, sample and variant call rate ≥ 0.98 and *p* ≥ 0.0001 from an exact test of HWE while omitting genomic regions with long-range linkage disequilibrium (LD)^65^. Genotyped data were later imputed to Phase 3, cosmopolitan reference option, of the 1000 Genomes Project panel using the University of Michigan Imputation server^66^ which implements minimac3^67^. Following imputation, all markers with imputation quality score *R*^2^ < 0.6 and MAF < 0.05 in our population were removed. Samples were processed and typed for the Illumina Methylation450 chip by the CWRU School of Medicine Genomics Core.

Principal components (PC) obtained from PCA and the genetic relationship matrix (GRM) were generated using genotyped markers that met QC criteria. We used PC-AiR and PC-Relate from the Bioconductor package GENESIS version 2.6.1^68^ to generate our PCs and GRM, respectively. PC-AiR accounts for sample relatedness to provide ancestry inference that is not confounded by family structure, while PC-Relate uses the ancestry representative PCs from PC-AiR to provide relatedness estimates due only to the recent family (pedigree) structure.

To examine cross-trait correlation, we used GCTA version 1.24.4^69^ to run a bivariate REML analysis for each pair of tests and tested for genetic correlations equal to 0. GCTA’s bivariate REML analysis estimates the genetic variance of each test and the genetic covariance between the two tests that can be captured by all SNPs^70^. Here we included all SNPs with MAF ≥ 0.01. The genetic variance/covariance calculated was adjusted for sex and the first two PCs.

We used RVTests, version 2.0^71^ to conduct our GWAS for each of the 16 communication phenotypes, assuming an additive effect of alleles and restricting to all common SNPs with MAF > 0.05. Phenotypes were transformed using a Box-Cox transformation (MASS, R) when applicable (Supplementary Table 2). We specifically relied on RVTest’s Grammar-gamma test^72^, which performs a linear mixed model association test while allowing for genotype dosages and accounting for family structure using the GRM. Because each of our tests was age-normed we included only sex and the first two PCs as covariates in our regression models.

### PRS analysis

In addition, we generated endophenotype-based PRS in the European subset of the CFSRS where genotype data, as well as clinical group data (no disorder, SSD only, language impairment (LI) only, SSD + LI, CAS) were available. This analysis was done to elucidate the connection between the genetic architecture of these endophenotypes and standard clinical diagnosis seen in clinics. Risk scores were derived from association statistics from our CFSRS GWASs and were constructed using PLINK 1.9^73^ (clump and score functions). Regions were considered if at least one variant in the region met the threshold for inclusion as a risk variant (*p* < 0.001). Clumping of variants was done in selected regions around the variant showing the strongest association in the region, removing other variants in linkage disequilibrium (*r*^2^ > 0.5). We used a linear mixed model to model the relationship between PRS and clinical group, controlling for sex and familial relationship (based on family ID*)*. Nested model comparison (the full model with the clinical group included versus the reduced model with clinical group removed) using the chi-squared test was implemented to determine if the clinical group explained a significant amount of variability in polygenic risk. These PRSs were used to examine the hypothesis that an increase in PRS score would associate with more complex clinical phenotypes when comparing SSD only versus SSD + LI and CAS.

### Statistical analysis of methylome-wide data

Quality control and normalization of raw methylation data (as Illumina.idat files) were carried out using the Bioconductor package RnBeads version 2.3.3 for R^74^. We removed methylation probes in non-CpG contexts, with nearby SNPs, on the X and Y chromosomes, and probes with low variability (SD < 0.005), leaving a total of 470,870 CpG markers with detection *p* value < 0.05. We normalized signal intensity by means of the BMIQ algorithm^75^, which adjusts for differences between Infinium I and II loci, and adjusted background by the methylumi NOOB procedure, as implemented in RnBeads. Our final data set was scaled to proportion of methylated DNA strand (*β*) values. Duplicate pairs were verified through concordance of genotypes for 65 SNPs on the Methylation450 chip. The final data set typed for the Methylation450 panel comprised 713 unique individuals, plus 60 duplicate samples.

The source of DNA for the MWAS came from saliva samples. Because our sample included salivary DNA samples, we were unable to adjust for cell-type composition using a blood-sample-based reference. Instead, we conducted principal components analysis (PCA) on genomewide methylation as follows: We selected 287,720 CpG sites with SD ≥ 0.02 across the entire sample and normalized the beta values for each site to mean = 0, SD = 1, creating an *m* × *n* matrix X, where *m* is the number of markers and *n* the number of samples. The eigenvectors from the matrix X′X/(*m* − 1), an *n* × n matrix, were obtained using the eigen() function in R, to be used as PC covariates in methylome-wide association studies (MWAS). We regressed our SSD outcomes on each of the first 20 PCs, and included significantly associated PCs in MWAS. Phenotypes were adjusted for between one and four PCs.

We tested for association between CpG beta values and endophenotypes using the linear mixed model approach of GRAMMAR-Gamma^72^ as implemented in RVtests^71^. Because our phenotypes were age-normed, we did not adjust for age, but rather for sex and one to four PCs.

We conducted a targeted cis-methylation QTL analysis over 521 CpG sites within 50 kilobasepairs (kb) of 162 candidate SNPs (Supplementary Data 1), using Matrix eQTL version 2.2^76^ to find the effect of genotype on the extent of methylation in a sample of 597 individuals with both epigenetic and imputed genotype data. All pairs of SNPs and CpG sites within 100 kb were considered to be in cis. Methylation was expressed as *M* values, where *M* = log(*β*/(1 − *β*)), which extends the range of possible values to (−∞,∞), making the values suitable as an outcome for linear regression.

### Replication dataset—ALSPAC

To replicate our GWAS findings, we obtained data from the Avon Longitudinal Study of Parents and Children (ALSPAC). The ALSPAC study was a prospective population-based birth cohort of babies born from >14,000 pregnancies between April 1991–December 1992, who were followed prospectively with a wide battery of developmental tests, parental questionnaires, child-completed questionnaires, and health outcomes^77–79^. Pregnant women residents in Avon, the UK with expected dates of delivery from 1st April 1991 to 31st December 1992 were invited to take part in the study. The initial number of pregnancies enrolled is 14,541 (for these at least one questionnaire has been returned or a “Children in Focus” clinic had been attended by 19/07/99). Of these initial pregnancies, there was a total of 14,676 fetuses, resulting in 14,062 live births and 13,988 children who were alive at 1 year of age. The study website contains details of all the data that is available through a fully searchable data dictionary (http://www.bris.ac.uk/alspac/researchers/data-access/data-dictionary). Blood samples were also collected for biomarker and genetic analyses.

Ethical approval for the study was obtained from the ALSPAC Ethics and Law Committee and Institutional Review Board of Case Medical Center and University Hospitals. Because this was a birth cohort, all children were included, regardless of diagnosis. We obtained both parental report data on speech development in the children, and also communication measures similar to those that we analyzed (see Communication Measures above and Supplementary Table 3). As this was a longitudinal study, different measures were given at different ages, and when the same domain was tested at two different ages, the identical measure was not used. At some ages, only random subsets were selected, so the sample size available from each age is not the same. In Supplementary Table 3, we list the measures given in the CFSRS battery along with the most similar measure given in ALSPAC. Because all the children were the same age when specific assessments were given, no age adjustment was needed. There were no equivalent measures for RAN and Elision.

Genotype QC was performed previously by ALSPAC^16^. We restricted our ALSPAC sample to unrelated individuals by randomly removing one from a pair of twins, when applicable. PCs were generated using Hail 0.1 software, to accommodate the format of files obtained from ALSPAC, using a standard PCA approach^80^. In generating the PCs we first removed long-range LD regions and restricted to variants with a MAF > 0.01, an imputation quality score of >0.95, and variants not in LD (*r*^2^ < 0.2; following the same process as with PLINK’s –indep-pairwise default procedure). Genetic association testing was performed using linear regression in Hail 0.1 when outcome measures were continuous and using logistic regression in Hail 0.1 when outcome measures were binary. We restricted our analyses to variants with a MAF > 0.01 and an imputation quality score of >0.6; we used a lower MAF threshold because we hypothesized that causal variants might be rarer in a population-based cohort compared to a cohort that was ascertained through a trait of interest. Covariates adjusted for included sex and the first two PCs. Age was not a consideration as ALSPAC is a longitudinal birth cohort study and age differences were negligible for any given measure.

### Functional annotation and results integration

In this analysis, we considered CFSRS the discovery sample, since families were ascertained through a child with SSD, and used ALSPAC as the replication sample. We identified associated loci with SNPs significant at *p* < 10^−5^ in CFSRS and *p* < 0.05 in ALSPAC, with effects in the same direction.

Because the majority of our findings are intergenic and/or fall in noncoding regions, we relied on annotation tools FUMA version 1.35d and HaploReg to characterize which genes our variants might affect, as well as variants’ functionality. We utilized FUMA^81^ for mapping genes to our variants based on genomic proximity, eQTL evidence, and chromatin interactions evidence. Default settings in FUMA were used, with the exception of tissue specificity. We hypothesized that gene expression and regulation would be most relevant in the brain and neural tissues, as well as muscles related to speech. In FUMA, we focused on eQTL and chromatin interaction evidence in our target tissues (brain and muscle). Additional details are found in the Supplement. HaploReg v.4.1 was used to examine the chromatin state evidence predicting whether the variant fell in a promoter or enhancer region. Using HaploReg v4.1 we examined histone marks indicating enhancer/promoter for brain tissues, neural tissues (including neuronal progenitor cells) and skeletal muscle tissue.

In order to further prioritize and synthesize our findings, we annotated associated loci as described above, including annotation of associated effects of these loci in the literature, and incorporated supportive findings from our MWAS (Supplementary Data 1). We generated a simple locus priority score as the sum of the number of times a locus included an enhancer and/or promoter, included an eQTL, was previously associated with a communication disorder and/or neuropsychiatric disorder, showed eQTL or chromatin state evidence specific to brain and/or neural tissues, mapped to a gene that was a *FOXP2* target in brain tissue^34–36^, and an meQTL in that region (at *p* < 5 × 10^−5^) with an associated methylation site (at *p* < 0.05) with the same phenotype as the associated GWAS loci, as determined using the bioinformatic resources described above.

We applied the EpiXcan pipeline^82^ to train gene expression predictors in human brain tissue. For genotypes and gene expression, we used psychENCODE data from the dorsolateral prefrontal cortex (DLPFC)^83^. We restricted our analysis to 924 Caucasian samples. We initially computed eQTL summary statistics using the R package Matrix eQTL version 2.2^76^, followed by estimation of SNP priors through the qtlBHM Bayesian hierarchical model^84^ using the Roadmap Epigenomics Project chromatin states for DLPFC (‘BRN_DL_PRFRNTL_CRTX’). In total, 363,955 predictors for 18,425 genes were recruited in the EpiXcan psychENCODE model. We then applied the S-PrediXcan method^85^ using the EpiXcan psychENCODE model as well as the SNP covariance matrix on the GWAS summary statistics. These analyses were based on genome-wide association results from two phenotypes from our GWAS, TWS, and Elision; these traits were chosen because they had the greatest number of unique significantly associated loci. Detailed results are in Supplementary Tables 7 and 8.

Chromatin interaction mapping was performed in FUMA using Hi-C data from PsychENCODE^83^ (Hi-C based enhancer-promoter interactions), Schmitt et al.^86^ (Hi-C based (significant loops) of cell line GSE87112, tissues Dorsolateral Prefrontal Cortex, Hippocampus and Neural progenitor cell) and Giusti-Rodriguez et al.^87^ (Hi-C data (significant loops after Bonferroni correction (Pbon < 0.001)) of adult and fetal cortex). Chromatin interactions were filtered by FDR < 1 × 10^−6^.

We primarily focused on loci with priority scores >5, and for loci with priority scores equal to 5, we examined loci with compelling evidence in the communication disorders literature and/or our own methylation data. These loci were then examined using colocalization analysis in LocusFocus^88^, as described below, which facilitates the exploration of a GWAS signal and the degree of colocalization with eQTLs in relevant tissue.

We used LocusFocus version 1.4.9^88^ to explore our GWAS signals in their degree of colocalization with expression quantitative trait loci (eQTL) for genes within ±200 kb of the lead SNP in the relevant GTEx tissues. The aim of this method is to annotate GWAS-derived associations to the most probable gene(s) and tissue(s) that may be driving that signal. This method uses the Simple Sum method to assess the degree of colocalization of any two given datasets. The Simple Sum region used for calculating colocalization of eQTLs and GWAS signals is ±100 kb of the lead SNP (i.e., GWAS signals and eQTLs within 100 kb of the lead SNP for genes within 200 kb of lead SNP). When applied to GTEx, LocusFocus presents the degree of colocalization of genes nearby the GWAS association for all the tissues selected in an interactive heatmap plot. Here we selected 14 tissues, including all brain tissue available for GTEx v7 (brain_spinal_cord_cervical, nucleus_accumbens_basal_ganglia, cerebellar_hemisphere, hippocampus, caudate_basal_ganglia, anterior_cingulate_cortex, cortex, hypothalamus, amygdala, frontal_cortex, substantia_nigra, putamen_basal_ganglia, cerebellum), as well as, skeletal_muscle. We also brought in psychEncode eQTL data^83^ (FDR < 0.05 and a filter requiring genes to have an expression > 0.1 fragments per KB per million reads (FPKM) in at least 10 samples) in as a secondary dataset to examine colocalization with our GWAS signal and eQTLs within psychEncode data. Here, we pulled eQTLs, within 100 kb of our lead SNP for genes within 200 kb of our lead SNP.

### Examination of previously identified candidate genes for communication disorders

In order to examine whether our GWAS replicated previous findings (either from published GWAS in language and reading phenotypes and/or targeted candidate gene studies of these phenotypes), we took a twofold approach. If the original papers provided rs IDs, we looked up our results at those specific SNPs. If the papers did not provide that level of detail, we instead examined all SNPs with MAF > 5% in the gene regions ±5 kb.

### Reporting summary

Further information on research design is available in the Nature Research Reporting Summary linked to this article.

## Supplementary information

Supplementary Information

Supplementary Data 1

Supplementary Data 2

Supplementary Data 3

Reporting Summary

## Data Availability

Data from the Cleveland Family Speech and Reading study are not available for broad genetic data sharing because study subjects did not provide informed consent for such data sharing, over 80% specifically saying that they wanted to be recontacted for additional use of the data. The IRB governing this study has imposed a restriction stating that the consent forms did not adequately cover the issue of deposition of the data into public repositories and that participants needed to be reconsented. In an effort to reconsent them, we have attempted to recontact these participants on numerous occasions, but have only been marginally successful. Summary statistics are not provided because of concerns that subjects can be identified from summary statistics, based on published literature demonstrating this is possible, and because these phenotypes are sufficiently rare and participants were ascertained in a narrow geographic region. Please contact the corresponding author, Sudha Iyengar, ski@case.edu, to request summary statistics. These can be shared on request but will require an IRB application, and submission of names of individuals who will use the data to our IRB.

## Code availability

All software versions are identified within the Methods. If there is no version number, then that software package only has one (current) version. There were no custom scripts created for the analyses conducted in this paper. Only one variable was created in this analysis (DDK) and those methods are fully described with the Measures.
