# Supplementary material for: Association between genes regulating neural pathways for quantitative traits of speech and language disorders
Source: NPJ Genom Med. 2021 Jul 27;6:64. doi: 10.1038/s41525-021-00225-5 (PMC8316336; doi:10.1038/s41525-021-00225-5)

SUPPLEMENTARY NOTE

Here we provide additional details on the measures given within the two studies. All other analytical methods are provided in the main manuscript document. Citations and short descriptions of these measures are also provided in the main manuscript document.

Measures – Cleveland Family Speech and Reading Study (CFSRS)

Oral Motor Skills Test

The *Robbins and Klee Oral Speech Motor Control Protocol* [1] (DDK-OSMCP) assessed oral motor skills in children 4 to 6 years. Diadochokinetic rates on single, double and multi-syllables were timed and scored. The *Fletcher Time-by-Count Test of Diadochokinetic Syllable Rate* [2] (DDK-FL) was administered to children 7 years or older. This test also assesses rates of syllable repetition on single, double and multi-syllable sequences. Z-scores were generated, and the scores for single and multi-syllables were combined for analysis. A shorter time for syllable sequence repetition indicates better oral motor skills. Because the DDK-OSMCP is scored in the opposite direction, these values were negated prior to merging with the DDK-FL; for this merged score, lower scores imply worse performance. We shall refer to this merged variable as DDK.

Expressive and Receptive Vocabulary

The *Expressive One Word Picture Vocabulary Test-Revised* (*EOWPVT* [3]) assesses expressive vocabulary and requires the examinee to name pictures representing objects, actions, and concepts. The *Peabody Picture Vocabulary Test- Third Edition (PPVT-III* [4]) tests receptive vocabulary and requires the examinee to point to the image that best matches the stimulus from a group of four. Both of these measures are given to children ages 2 years or older.

Multisyllabic Nonword and Real Word Repetition

Nonsense word repetition (NSW [5]) requires children to repeat 15 non-words, a task which requires encoding of unfamiliar phonological sequences; deficits in encoding would result in inaccurate word repetition[5] and can discriminate children and adults with resolved SSD from those who never had SSD [6]. While this task is normally given to individuals ages 4 years through adulthood, the children with CAS generally could not perform this task in preschool, so the first available assessment starting at age 7 was used for this analysis. The Multisyllabic Word Repetition task (MSW) requires children to accurately sequence phonemes by repeating real multisyllabic words. Target words include aluminum, thermometer, and sympathize [5]. The test is scored by determining the percentage of words repeated correctly. In addition, we scored the percentage of phonemes repeated correctly; these tests are the MSW-PPC and NSW-PPC.

Phonological Awareness Measure

The *Elision* subtest of the *Comprehensive Test of Phonological Processing* [7] requires the individual to repeat a word and then say the word with a sound or syllable deleted. For example, "Say the word tulip. Now say it without the "tu". The Elision subtest is a measure of phonological awareness, that is knowledge of the sound system of oral language. Children with poor phonological awareness skills demonstrate weakness in single word reading. In addition, we gave an earlier version of the Elision task, Comprehensive Test of Phonological Processing-Experimental Version (Wagner, Torgesen, & Rashotte, 1994, personal communication), which we combined with this CTOPP subtest in order to create a single variable for analysis. We transformed the CTOPP Elision subtest into a z-score prior to merging with the original Elision.

Rapid Automatized Naming

The Rapid Color Naming (RAN) requires the individual to name colored squares as quickly as possible. The total number of seconds to name all the colors is the individual's score. This test measures the subject's ability to retrieve phonological information from long-term memory and is

predictive of reading decoding skills. We had an older version of this task[8] as well as the CTOPP subtest. We transformed the CTOPP into a z-score and flipped (or negated) the RAN prior to merging CTOPP colors, a scaled score with a mean of 10 and standard deviation of 3, and RAN colors, a z-score with larger values representing worse performance.

Spoken Language Measures

There were two language assessments that were combined, Clinical Evaluation of Language Fundamentals- Revised (CELF-R) [9] and the Test of Language Development- Primary- Second Edition (TOLD-P2) [10].   For pre-school age children, the equivalent subtests on the Clinical Evaluation of Language Fundamentals-Preschool was given. Participants enrolled in the study after the release of a newer version of the tests were given the new version. Both the CELF-R and TOLD-P2 provide standard scores for receptive and expressive language skills. We merged the CELF-R receptive with the TOLD-P2 listening quotient, with preference to the CELF-R if both were given at the same time point. Both the CELF-R and the TOLD-P2 were scaled scores, with a population mean of 100 and standard deviation of 15. We merged the CELF-R expressive with the TOLD-P2 speaking quotient, with preference to the CELF-R if both were given at the same time point. Both the CELF-R and the TOLD-P2 were scaled scores, with a population mean of 100 and standard deviation of 15.

Reading Measures

The *Woodcock Reading Mastery Test-Revised, Word Attack subtest (WRMT-AT [11])* evaluates phonetic decoding skills by requiring examinees to read a list of 45 non-words; the test includes nonwords such as *ip, din, ceisminadolt, and gnouthe*. The *Woodcock Reading Mastery Test-Revised, Word Identification Subtest (WRMT-ID [11])* assesses single word reading ability by requiring examinees to read a list of 106 real words. This task is given to individuals ages 5 through adulthood. The *Wechsler Individual Achievement Test* [12] has 2 subtests. The *Reading*

Comprehension subtest (WIAT-RC) consists of printed passages that the individual reads and then answers orally presented question on the passage. It assesses the individual's ability to recognize details and make inferences concerning what has been read. The reading comprehension subtests provides information on the child's ability to use picture cues, recognize detail, sequence events, identify cause-effect relationships, make inferences, and compare and contrast characters, objects or events from the passage. The *Listening Comprehension* subtest (WIAT-LC) consists of orally presented passages that are sometimes paired with pictures. The child understanding of the passage is examined by answering questions about the passage. The Listening Comprehension subtest, similar to the Reading Comprehension Subtest, assesses the child's ability to use picture cues, recognize detail, sequence events, identify cause-effect relationships, make inferences, and compare and contrast characters, objects or events from the passage.

ALSPAC Study Communication measures

We worked with the ALSPAC team to find the most equivalent measures to match those in the CFSRS. These are summarized in Supplementary Table 3. The *multisyllabic word repetition (MWR)* task consisted of repeating "buttercup" and dinosaur" 5 times each – the total correct score was used for analysis. This task was given at age 5. A *nonsense word repetition task* [13] *(CNrep)* was given at age 5 (*CNrep5*), and a shortened version of this task was given at age 8 (*CNrep8)*. Two reading tasks were given. At age 7, both the *Wechsler Objective Reading Dimensions single word reading task (WORD)* and *Neale analysis of reading ability (NARA)* were given. The NARA has a reading comprehension (*NARA-C*) and reading accuracy (*NARA-A*) subtest. A non-word reading task was designed specifically for ALSPAC, so we refer to it as *ALSPACread*. The spelling tasks were based on work by Nunes et al.[14], so we refer to this task as *ALSPACspell7* and *ALSPACspell9* for the tests given at ages 7 and 9, respectively. The

Wechsler Objective Language Dimensions (*WOLD)* is a test of expressive language ability in children, and has several subtests that were used for language and vocabulary assessment: *comprehension (WOLD-C*), *expression (WOLD-E)*, and *vocabulary (WOLD-V)*; these were all given at age 8.

1 Robbins, J. & Klee, T. Clinical assessment of oropharyngeal motor development in young children. *Journal of Speech and Hearing Research* **52**, 271-277 (1987).

2 Fletcher, D. The Fletcher time-by-count test of diadochokinetic syllable rate (C.C. Publications, Inc., Tigard, OR, 1977).

3 Gardner, M. Expressive one word picture vocabulary test-revised. (Academic Therapy Publications, Novato, CA, 1990).

4 Dunn, L. & Dunn, L. Peabody picture vocabulary test - third edition (American Guidance Service, Inc, Circle Pines, MN, 1997).

5 Catts, H. Speech production/phonological deficits in reading disordered children. *Journal of Learning Disabilities* **19**, 504-508 (1986).

6 Lewis, B. A. *et al.* Speech and language skills of parents of children with speech sound disorders. *Am J Speech Lang Pathol* **16**, 108-118 (2007).

7 Wagner, R. T., J; Rashotte, C; Pearson, NA. Comprehensive Test of Phonological Processing (Pearson, London, England, 2013).

8 Denckla, M. R., RG;. Rapid automatized naming of pictured objects, colors, letters and numbers by normal children. *Cortex* **10**, 186-202 (1974).

9 E, S., Wiig, E. & Secord, W. *Clinical evaluation of language fundamentals-Revised*. (The Psychological Corporation, 1987).

10 Newcomer, P. & Hammill, D. *Test of language development - Primary, Second Edition*. (Pro-Ed., 1988).

11 Woodcock, R. Woodcock Reading Mastery Test. (American Guidance Service, Circle Pines, MN, 1987).

12 Wechsler, D. Wecshler intelligence scale for children-third edition (The Psychological Coporation, San Antonio, TX, 1991).

13 Speech production/phonological deficits in reading disordered children. *Journal of Learning Disabilities* **19**, 504-508 (1986)

14 Nunes, T. B., P; Olsson, J; . Learning Morphological and Phonological Spelling Rules: An Interventinoal Study. *Scientific Studies of Reading* **7**, 289-307 (2009).

SUPPLEMENTARY TABLES

Supplementary Table 1. Age distribution and binary trait prevalence for CFSRS measures

Test	N	Mean age (SD) [range]	% Female	% Lang	% SSD	% CAS
NSW_PPC	431	14.0 (12.4) [4, 64]	45%	30%	54%	11%
NSW	431	14.0 (12.4) [4, 64]	45%	30%	54%	11%
MSW_PPC	432	14.0 (12.4) [4, 64]	45%	30%	55%	11%
MSW	432	14.0 (12.4) [4, 64]	45%	30%	55%	11%
PPVT	399	12.5 (12.0) [2.5, 64]	44%	32%	57%	12%
EOWPVT	364	10.4 (9.6) [2.5, 64]	42%	33%	59%	13%
WRMT-ID	399	16.4 (11.7) [5, 64]	45%	29%	53%	10%
WRMT-AT	398	16.4 (11.7) [5, 64]	45%	29%	53%	10%
TWS	298	10.3 (2.4) [5, 18]	40%	33%	59%	12%
Elision	299	10.4 (3.7) [4, 22]	40%	35%	62%	14%
RAN	309	9.0 (3.9) [4, 23]	40%	33%	62%	14%
CELF_Receptive	325	7.4 (3.1) [3, 18]	40%	34%	62%	13%
CELF_Expressive	325	7.4 (3.1) [3, 18]	40%	34%	62%	13%
DDK	419	13.5 (12.7) [2.5, 64]	45%	29%	55%	11%
WIAT_LC	165	16.2 (11.9) [7, 51]	44%	27%	42%	3.70%
WIAT_RC	166	16.0 (11.9) [7, 51]	45%	28%	42%	3.60%

Each row specifies statistics for each of the 16 CFSRS measures. Percentages shown are based on the sample having available test data for the corresponding CFSRS measure (% out of N).

Supplementary Table 2. Descriptive statistics for CFSRS measures with Box-Cox transformation parameters when needed

Test	Mean	SD	Lambda, C
WIAT_RC	104.14	15.50	NA
WIAT_LC	108.02	15.60	NA
MSW_PPC_t	79.79	35.12	3.4; 4.9
NSW_PPC_t	38.27	17.23	3.0; 4.7
NSW	0.00	1.00	NA
MSW	0.00	1.00	NA
CELF_R	0.00	1.00	NA
CELF_E	0.00	1.00	NA
Elision_t	3.09	1.64	1.4; 3.1
RAN_t	310.06	89.76	3.2; 8.5
WRID_t	6.98	1.91	1.2; 4.3
WRAT_t	5.44	1.99	1.5; 4.3
TWS	0.95	0.89	NA
EOWPVT	0.00	1.00	NA
PPVT	0.00	1.00	NA
DDK_t	2928.78	1058.51	5.0; 6.6

"_t" appended to the end of the test variable name indicates the variable was transformed using a Box-Cox transformation, with corresponding lambda and shift parameter C shown. Means and SD reflect variable used (i.e., post transformation, if applicable).

Supplementary Table 3. Correspondence between CFSRS and ALSPAC measures

CFSRS measure	Corresponding ALSPAC measure(s)	Age given	Sample size for ALSPAC
Multisyllabic word repetition (MSW)	Repetition of “buttercup” and “dinosaur” (**MWR)**	5	711
Nonsense word repetition (NSW)	Children’s test of non-word repetition (**CNrep5**)<br>----<br>12 items from Children’s test of non-word repetition (**CNrep8**)	5<br><br>8	680<br><br>5860
Woodcock real-word reading (WRMT-ID)	WORD single word reading (**WORD**)<br>----<br>Neale analysis of reading ability (**NARA**) – reading comprehension (**NARA-C**) and reading accuracy subtests **(NARA-A**)	7	
Woodcock nonsense word reading (WRMT-AT)	Single word nonword reading **(ALSPACread)**	9	6136
Test of written spelling (TWS)	Single word spelling (**ALSPACspell7**)<br>-----<br>Single word spelling (**ALSPACspell9**)	7<br><br>9	6203<br><br>6137
CELF receptive (CELF_R)	WOLD comprehension (**WOLD-C**)	8	5865
CELF expressive (CELF_E)	WOLD expression (**WOLD-E**)	8	
Expressive one word vocabulary (EOWPVT)	WOLD expression – one word vocabulary test	8	5840
Peabody picture vocabulary (PPVT)	WISC vocabulary (**WISC-V**)	8	5844

Supplementary Table 4. Comorbidities in Probands

1	2	3+
50 (36%)	34 (25%)	53 (39%)

*Number of probands with 1, 2 or 3 or greater communication disorders (Apraxia, Speech, Language, Reading, Spelling).

Supplementary Table 5. Descriptive statistics for ALSPAC sample

Sample size (union across all tests)	9658
Age range	[5, 9]
Female N (%)	4773 (49%)
Speech problems (parent reported)	6%

Supplementary Table 6. Summary Statistics Table for Top GWAS hits in CFSRS

rsID	CHROM	POS	REF	ALT	N	AF	Beta	BetaVar	Pvalue	test
rs10793688	1	146988760	T	C	432	0.416	-0.316	2.12	6.77×10^{-6}	MSW
rs855865	1	159028378	C	T	431	0.806	-0.391	3.27	6.98×10^{-6}	NSW
rs10157401	1	30732871	G	A	419	0.142	-457.732	4293570	6.13×10^{-6}	DDK
rs3001844	1	55494735	T	C	165	0.423	-4.082	475.46	1.64×10^{-2}	WIAT_LC
rs74371111	10	46027420	A	G	397	0.076	1.376	32.63	1.62×10^{-6}	WAT
rs74879269	12	103677691	G	A	325	0.051	0.848	12.23	1.27×10^{-5}	CELF_E
rs11061229	12	131389783	G	C	298	0.055	0.845	10.52	7.23×10^{-6}	TWS
rs4762797	12	21002703	G	A	298	0.866	-0.500	3.65	6.49×10^{-6}	TWS
rs1231010	13	28329109	G	A	431	0.244	6.186	823.30	7.81×10^{-6}	NSW_PPC
rs1609593	13	79839523	T	C	325	0.392	-0.384	2.03	1.25×10^{-6}	CELF_E
rs57645874	14	35837476	A	G	299	0.428	0.433	2.36	1.10×10^{-6}	Elision
rs856379	14	59210646	A	G	432	0.928	0.614	7.49	3.16×10^{-6}	MSW
rs17736427	14	93195374	C	T	431	0.332	5.786	723.294	8.14×10^{-6}	NSW_PPC
rs7141746	14	94993936	G	A	165	0.851	-3.120	864.712	1.74×10^{-1}	WIAT_LC
rs1257267	14	99858970	C	A	397	0.383	0.701	9.595	6.58×10^{-6}	WRMT-AT
rs4888606	16	77231207	C	G	432	0.589	-0.316	2.198	9.59×10^{-6}	MSW
rs12953343	18	4023876	T	A	419	0.187	-424.939	3464380.000	2.96×10^{-6}	DDK
rs79681396	18	40822793	G	A	298	0.051	0.915	12.296	6.88×10^{-6}	TWS
rs8084940	18	56462735	A	G	165	0.143	10.561	927.781	8.85×10^{-6}	WIAT_LC
rs16848167	2	143378805	T	A	325	0.274	-0.421	2.762	5.03×10^{-6}	CELF_E
rs2601079	2	169280713	G	A	299	0.756	0.466	2.919	2.42×10^{-6}	Elision
rs1387088	3	1942898	T	C	431	0.498	-5.635	670.011	6.36×10^{-6}	NSW_PPC
rs150319190	3	39743136	T	A	325	0.155	-0.527	4.562	8.92×10^{-6}	CELF_R
rs2005865	4	27297733	G	A	299	0.126	-0.636	4.857	6.13×10^{-7}	Elision
rs16848539	4	73572756	T	A	432	0.054	0.707	10.050	3.58×10^{-6}	MSW
rs62300926	4	77531588	A	G	432	0.087	-0.546	6.469	8.11×10^{-6}	MSW
rs17691077	5	132043351	A	C	299	0.137	-0.616	5.324	4.02×10^{-6}	Elision

rs10475950	5	170102906	G	A	298	0.434	0.344	1.707	5.78×10^{-6}	TWS
rs2546671	5	172924967	C	T	325	0.482	0.414	2.219	5.62×10^{-7}	CELF_R
rs34651	5	72144005	C	T	165	0.945	-18.431	2479.900	2.10×10^{-6}	WIAT_LC
rs12673406	7	123604182	C	T	298	0.120	-0.540	4.309	7.45×10^{-6}	TWS
rs113748707	7	154706515	C	A	419	0.054	-594.826	11088300.000	2.56×10^{-4}	DDK
rs10122818	9	114335864	G	C	431	0.237	5.085	928.809	5.41×10^{-4}	NSW_PPC

For interpretation of betas (effect sizes) refer to Supplementary table 2 for means and standard errors of test variable.

Supplementary Table 7. PsychEncode EpiXcan method using Meta-analysis results of Elision GWAS

gene_name	zscore	effect_size	pvalue	var_g	pred_perf r2	pred_perf pval	pred_perf qval	n_snps used	n_snps in_cov	n_snps in_model	fdr
GTF2IP4	-4.39	-0.70	1.11E-05	0.01	0.01	3.67E-04	8.12E-04	14	17	15	0.14
CBWD2	4.01	0.13	5.97E-05	0.34	0.41	6.52E-117	3.53E-115	23	29	29	0.75
OTOA	-4.01	-0.78	6.17E-05	0.01	0.02	1.13E-05	2.81E-05	7	17	16	0.77
AC106788.1	3.97	0.97	7.27E-05	0.01	0.01	2.26E-02	4.35E-02	11	22	17	0.91
SLFN13	-3.65	-0.68	2.59E-04	0.01	0.02	1.80E-06	4.71E-06	11	18	12	1.00
SAP30L	-3.63	-0.33	2.89E-04	0.04	0.07	2.63E-15	1.13E-14	30	63	63	1.00
TANK	3.57	0.65	3.51E-04	0.01	0.01	2.89E-03	5.98E-03	14	23	22	1.00
NFKBIZ	-3.51	-0.49	4.52E-04	0.02	0.05	4.23E-12	1.55E-11	18	22	19	1.00
DNAJC25	3.50	1.14	4.73E-04	0.00	0.02	7.02E-05	1.64E-04	2	2	2	1.00
ANKRD52	-3.47	-3.59	5.27E-04	0.00	0.01	2.26E-03	4.72E-03	2	14	4	1.00

Supplementary Table 8. PsychEncode EpiXcan method using Meta-analysis results of TWS GWAS

gene_name	zscore	effect_size	pvalue	var_g	pred_perf r2	pred_perf pval	pred_perf qval	n_snps used	n_snps in_cov	n_snps in_model	fdr
SLC45A4	-4.18	-1.34	2.88E-05	0.01	0.01	2.07E-03	4.32E-03	8	30	24	0.36
SYCE1L	3.96	0.45	7.64E-05	0.08	0.10	4.50E-24	2.77E-23	23	29	29	0.95
NCBP2-AS2	-3.57	-0.36	3.55E-04	0.11	0.17	4.68E-39	4.67E-38	12	13	13	1.00
SENP5	-3.52	-2.17	4.28E-04	0.00	0.01	3.28E-04	7.29E-04	3	6	3	1.00
LINC01998	-3.51	-0.21	4.50E-04	0.22	0.31	3.78E-80	9.68E-79	4	6	4	1.00
PPP1R13B	3.49	1.69	4.85E-04	0.00	0.01	2.23E-04	5.02E-04	4	4	4	1.00
NCBP2	-3.41	-0.24	6.49E-04	0.19	0.23	4.18E-56	6.37E-55	55	100	89	1.00
IFI16	3.33	0.53	8.69E-04	0.04	0.16	4.38E-38	4.25E-37	21	39	34	1.00
ARPP21	3.33	1.04	8.77E-04	0.01	0.02	1.92E-04	4.35E-04	15	25	25	1.00
TCTN3	-3.32	-0.33	8.99E-04	0.08	0.11	2.87E-25	1.85E-24	20	33	29	1.00

Supplementary Data 1 – Results of methylation analysis of candidate gene regions

This file is supplied as a separate excel file (Supplementary Data 1.xlsx)

Supplementary Data 2. Association results from regions identified from published GWAS of reading and language phenotypes

This file is provided as a separate excel file named Supplementary Data 2.xlsx

Supplementary Data 3. Association results from regions identified from published GWAS of reading and language phenotypes

This file is provided as a separate excel file named Supplementary Data 3.xlsx

Supplementary Figures

Supplementary Figure 1. Distribution of associated SNPs in CFSRS

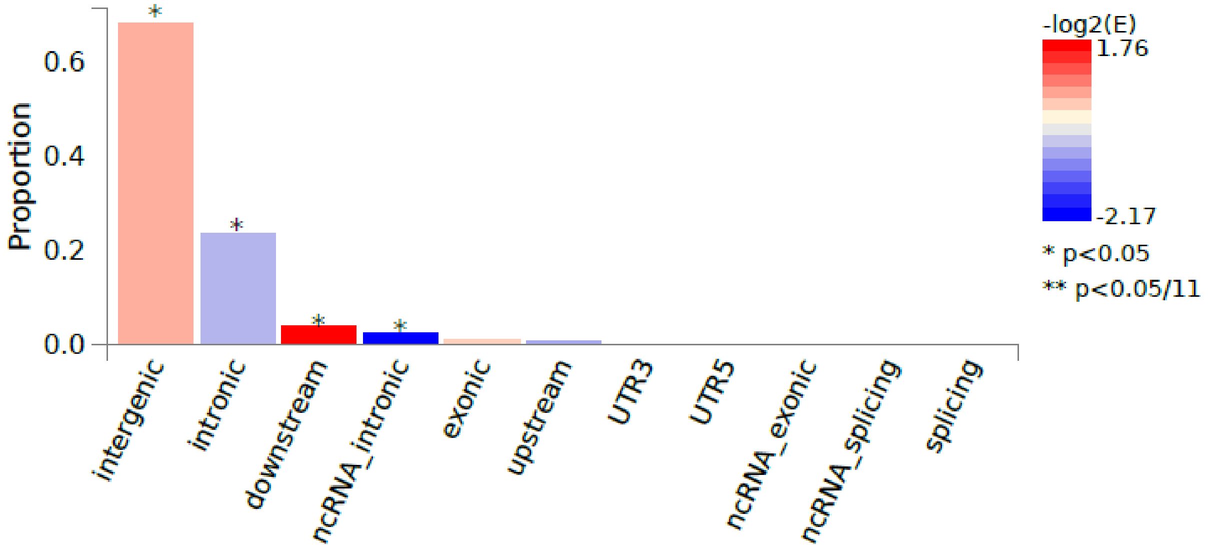

Supplementary Figure 2. Locus zoom plots and functional annotation for most signfiicant findings. Supplementary Figure 2 shows association results for the top loci. P-values displayed are for CFSRS and are for the test for which the top SNP was observed. Circles show P-values for SNP associations and triangles show P-values for methylation associations (specifically those for which the top SNP is a meQTL for). The larger plot shows the top SNP for each region +/- 200 kb. The window highlights the region that spans significant association results (P≤ 1×10-5 in CFSRS) in any CFSRS test. A. *IFI16* region (window spans chr1:159001292-159028378); B. *NFKBIA* region (window spans chr14:35770806-35846092); C. *DACT1* region (window spans chr14:59210335-59221002); D. *SETD3* region (window spans chr14:99858970-99942692). E. *MON1B* region (window spans chr16:77231207-77248555).

Supplementary Figure 2a

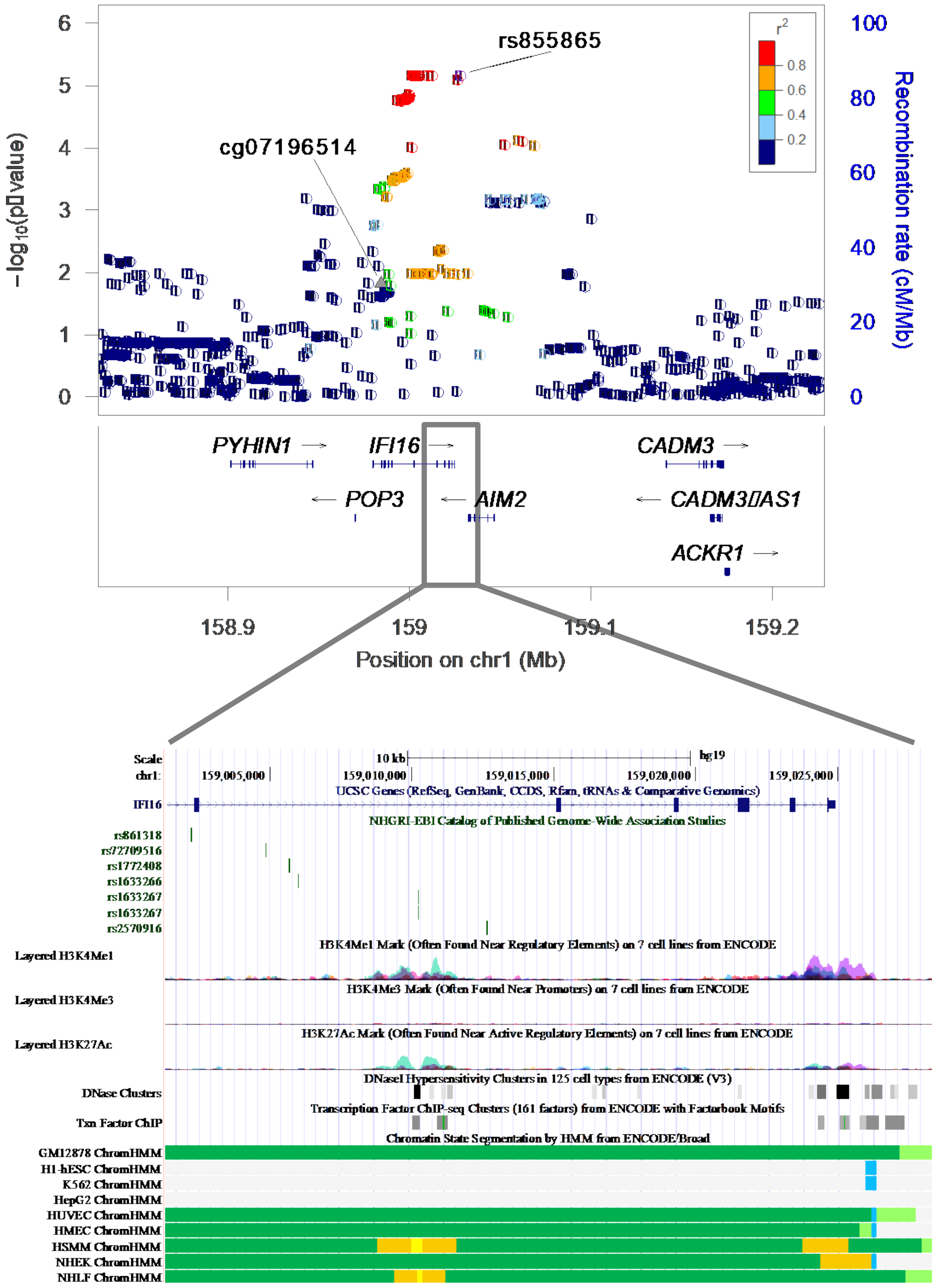

Supplementary Figure 2b.

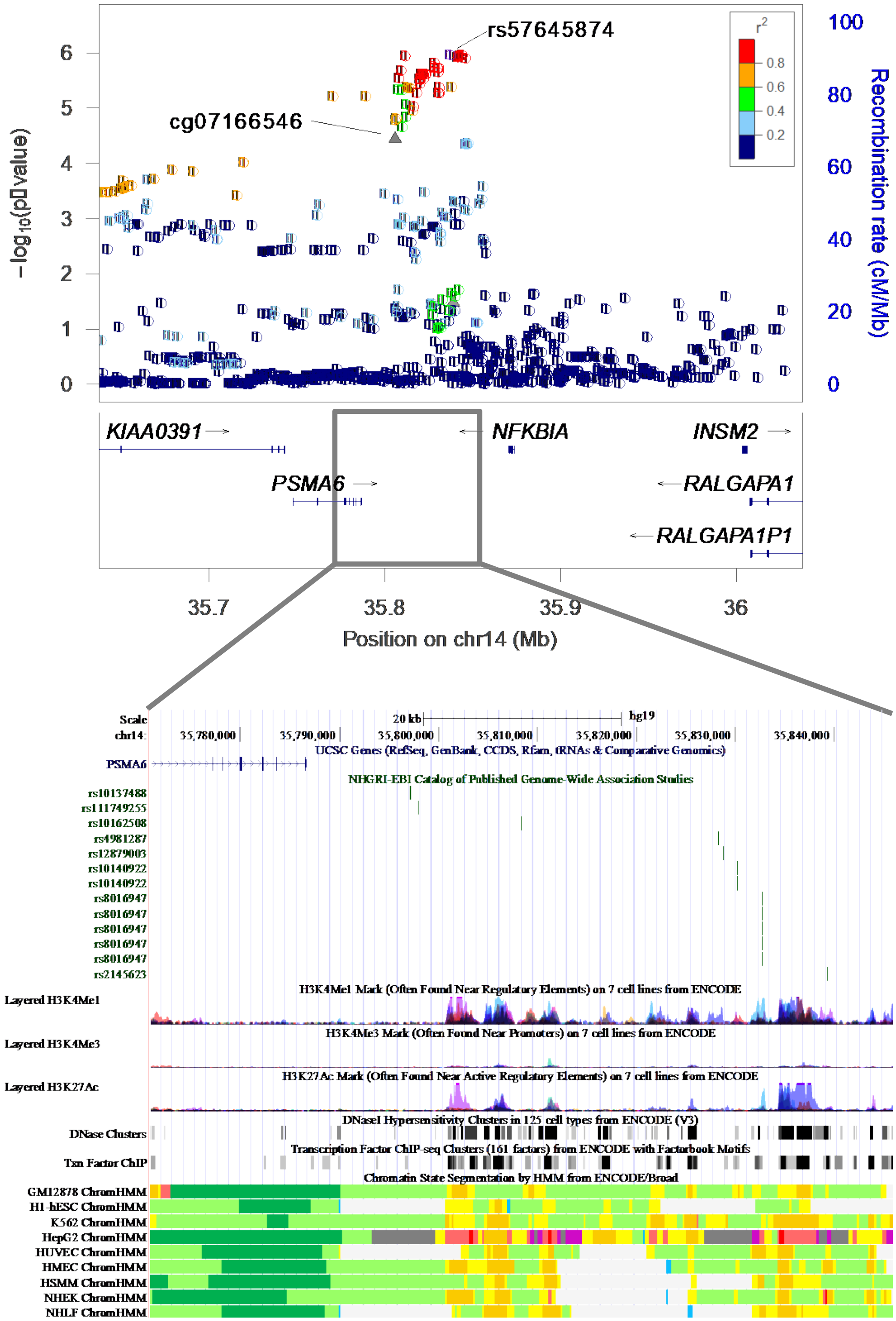

Supplementary Fig 2c

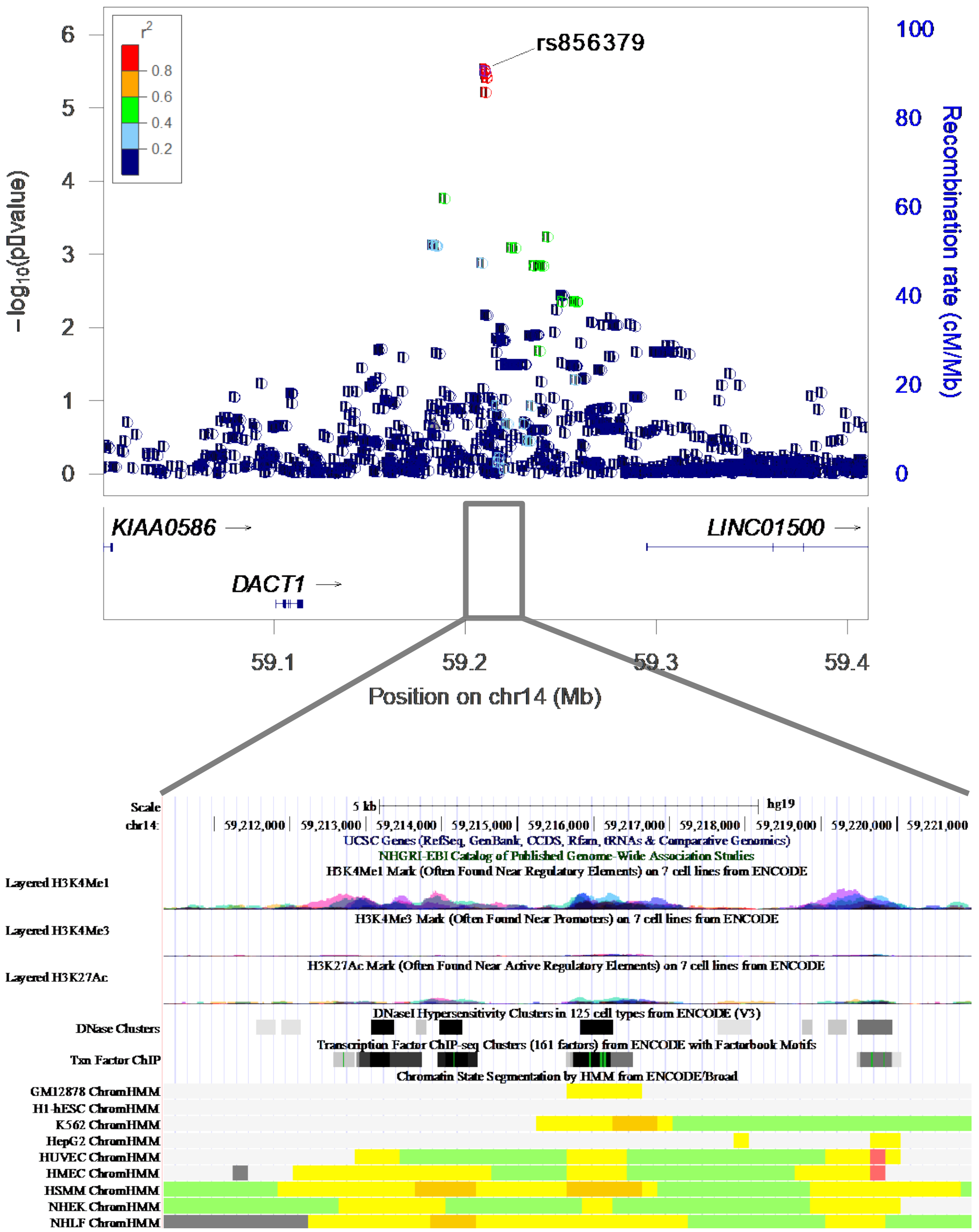

Supplementary Fig 2d

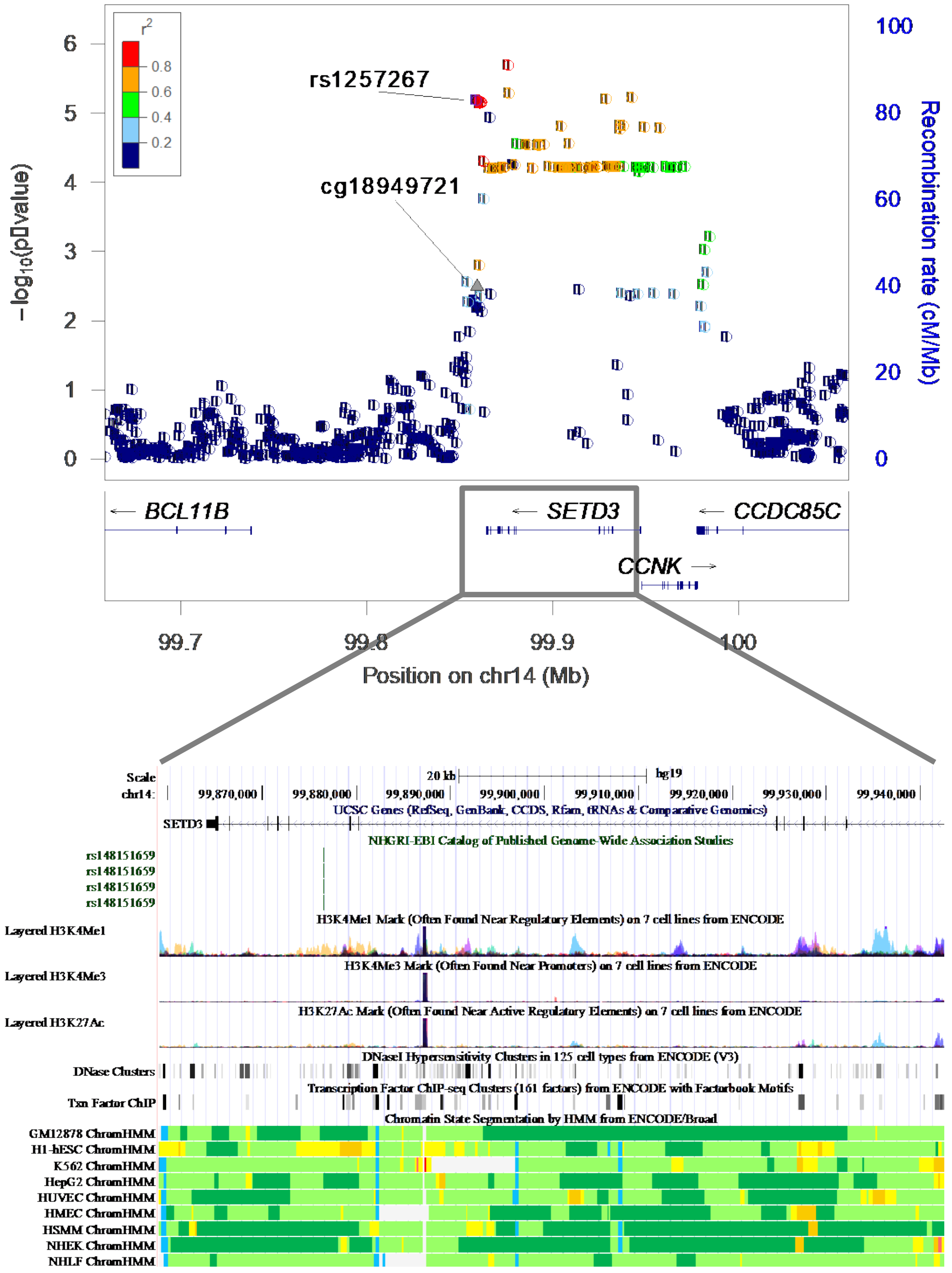

Supplementary Fig 2e.

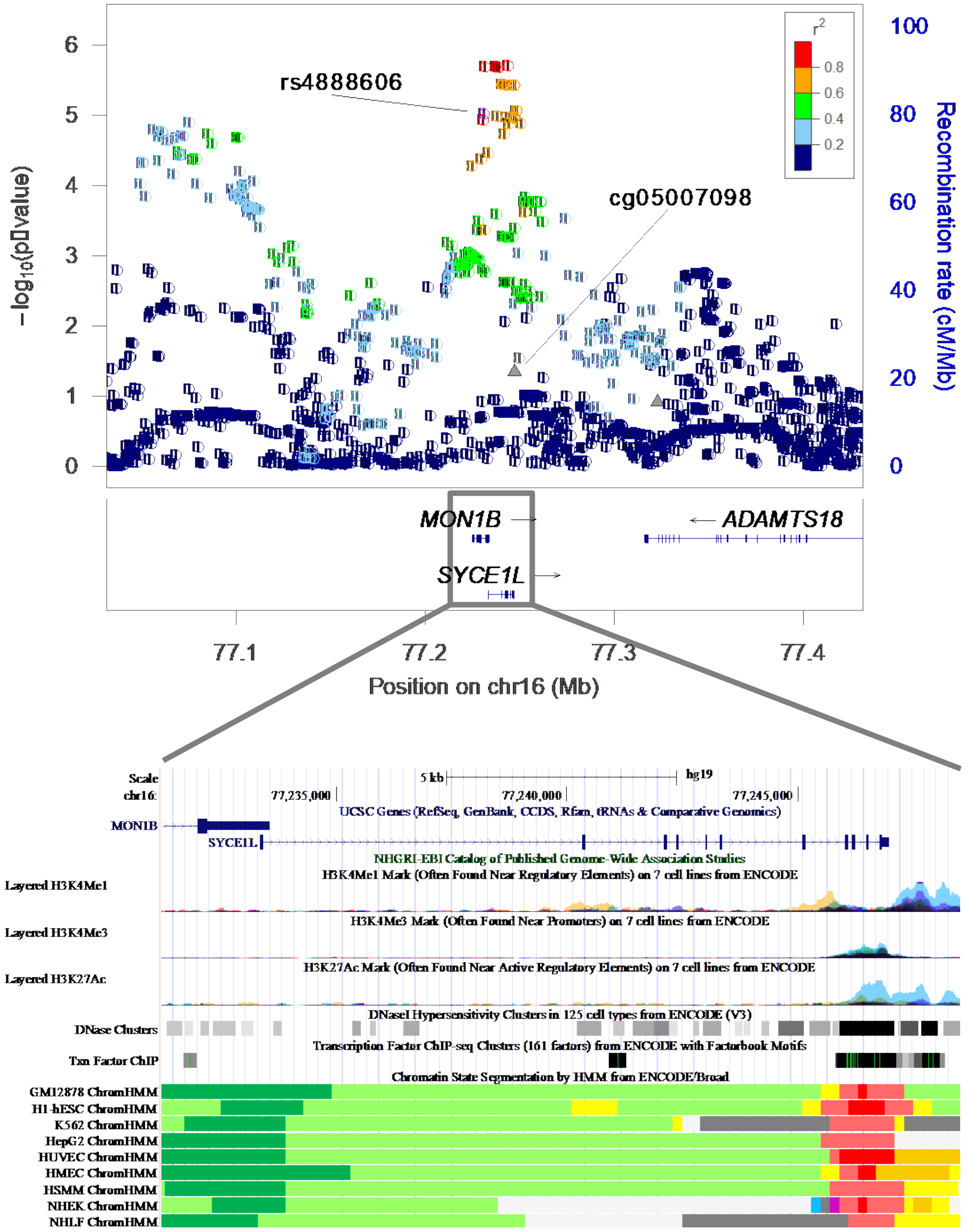

Supplementary Figure 3. Colocalization figures using LocusFocus. Each figure has 3 subfigures A-C. **A** Visualization plot depicts GWAS results for the associated speech communication phenotype as filled circles (corresponding y-axis on left) and eQTLs for the highlighted gene in GTEx tissues (spanning brain and skeletal muscle), as well as eQTLs for specified psychEncode genes as lines (corresponding y-axis on right). **B** Heat map shows Simple Sum colocalization results for each gene/tissue combination. Cell colors for gene-tissue pairs are based on strength of colocalization, as -$\log_{10}$(*SS* p-values) for that gene-tissue pair. Strength of colocalization is coloured from green (low -$\log_{10}$P) to red (high -$\log_{10}$P). **C** Colocalization results for PsychEncode eQTLs in specified genes in the region. Significant colocalization is determined by the suggested Simple Sum colocalization threshold (-log10 (0.05/#of tests)).

Supplementary Figure 3a. Colocalization Plots for *IFI16* region

A

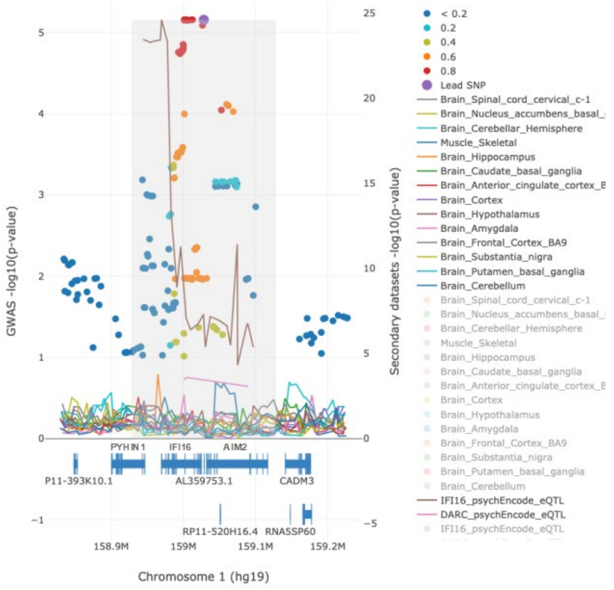

B

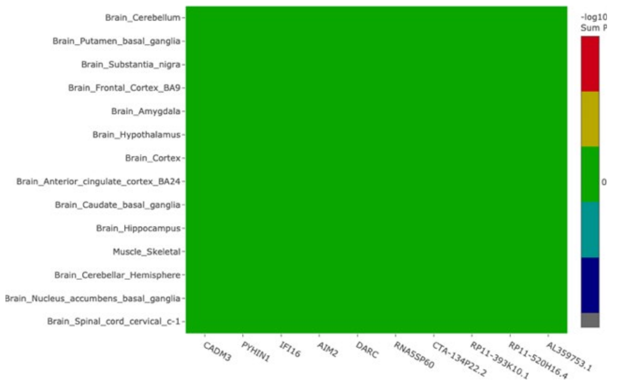

C

Dataset description	Simple Sum -log10P
DARC_psychEncode_eQTL	7.83e-02
IFI16_psychEncode_eQTL	5.44e-04

*Colocalization Plots for the *IFI16* region. The highlighted gene for which GTEx eQTLs are displayed in subfigure A is *IFI16* and the GWAS results are for NWR. Significant colocalization is determined by the suggested Simple Sum colocalization threshold of 1.6 (-log10 (0.05/#of tests)). PsychENCODE eQTL SS colocalization is not significant for the *IFI16* and *DARC* genes. For GTEx tissues there are no significant SS colocalizations seen for any gene/tissue combination in the region.

Supplementary Figure 3b. Colocalization Plots for *NFKBIA* region

A

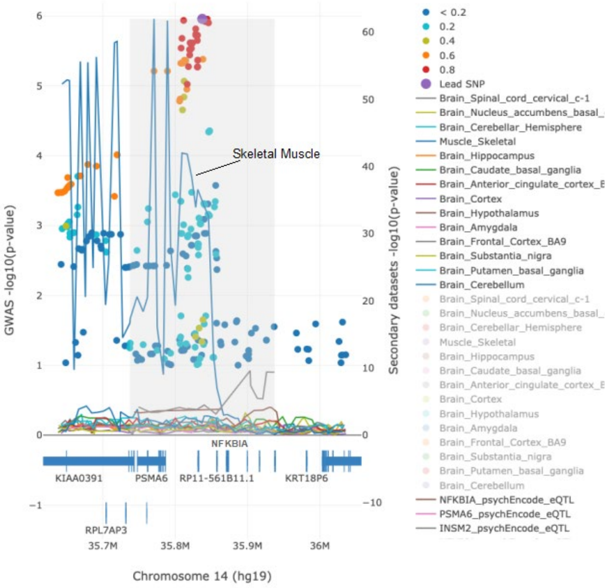

B

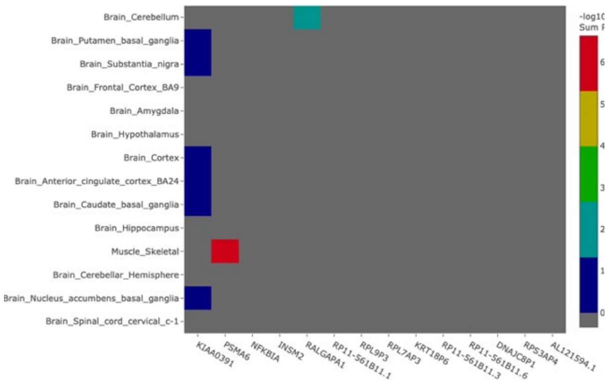

C

Dataset description	Simple Sum -log10P
INSM2_psychEncode_eQTL	7.73e-04
NFKBIA_psychEncode_eQTL	7.57e-01
PSMA6_psychEncode_eQTL	no significant eQTLs

*Colocalization Plots for the *NFKBIA* region. The highlighted gene for which GTEx eQTLs are displayed in subfigure A is *PSMA6* and the GWAS results are for Elision. Significant colocalization is determined by the suggested Simple Sum colocalization threshold of 2.2 (-log10 (0.05/#of tests)). PsychENCODE eQTL SS colocalization is not significant for the *NFKBIA*, *PSMA6* and *INSM2* genes. For GTEx tissues there is a significant SS colocalization seen for *PSMA6* in skeletal muscle.

Supplementary Figure 3c. Colocalization Plots for *DACT1* region

A

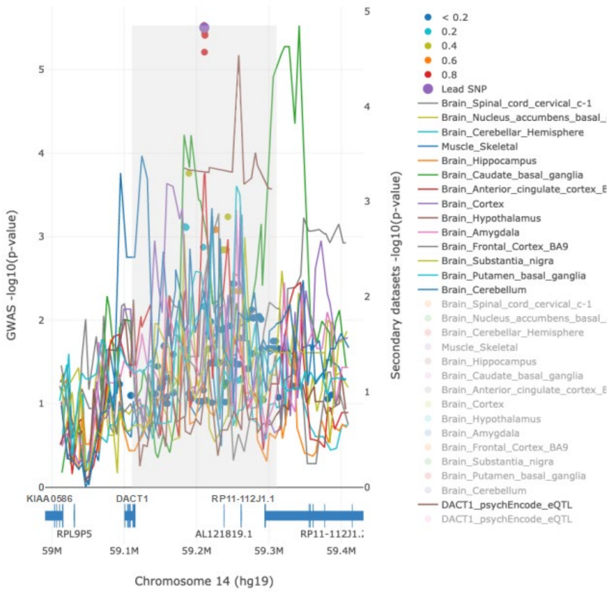

B

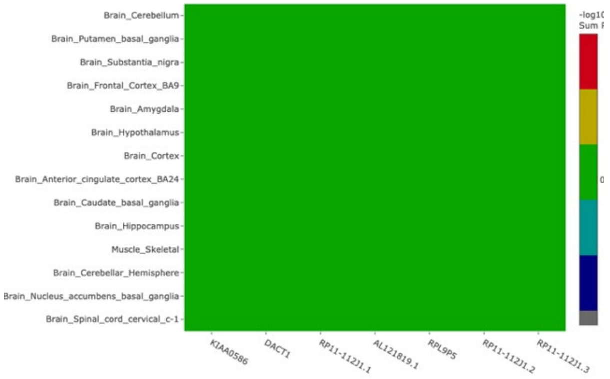

C

Dataset description	Simple Sum -log10P
DACT1_psychEncode_eQTL	1.24e-01

*Colocalization Plots for the *DACT1* region. The highlighted gene for which GTEx eQTLs are displayed in subfigure A is *DACT1* and the GWAS results are for MSW. Significant colocalization is determined by the suggested Simple Sum colocalization threshold of 1.3 (-log10 (0.05/#of tests)). PsychENCODE eQTL SS colocalization is not significant for the *DACT1* gene. For GTEx tissues there are no significant SS colocalizations seen for any gene/tissue combination in the region.

Supplementary Figure 3d. Colocalization Plots for *SETD3* region

A

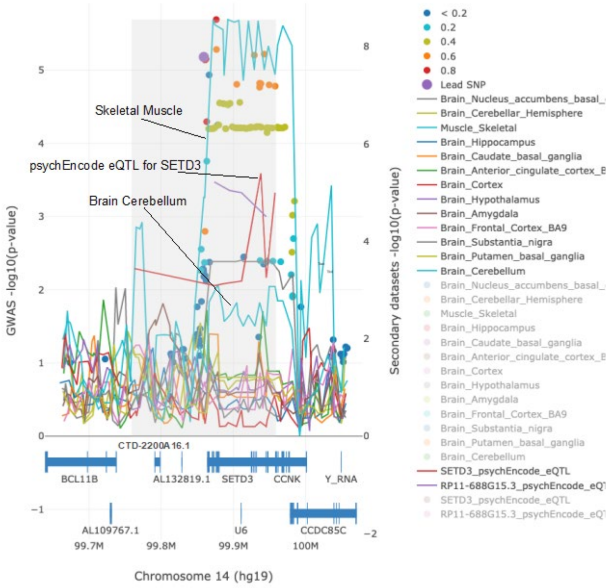

B

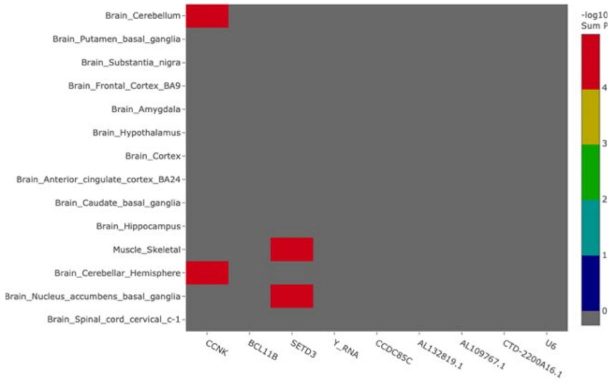

C

Dataset description	Simple Sum -log10P
RP11-688G15.3_psychEncode_eQTL	no eQTL data
SETD3_psychEncode_eQTL	2.66

*Colocalization Plots for the *SETD3* region. The highlighted gene for which GTEx eQTLs are displayed in subfigure A is *SETD3* and the GWAS results are for WRMT-AT. Significant colocalization is determined by the suggested Simple Sum colocalization threshold of 2.0 (-log10 (0.05/#of tests)). PsychENCODE eQTL SS colocalization is significant for the *SETD3* gene. For GTEx tissues significant SS colocalization is seen for *SETD3* in brain_nucleus_accumbens_basal_ganglia and skeletal muscle, and in *CCNK* in cerebellar hemisphere and cerebellum.

Supplementary Figure 3e. Colocalization Plots for *MON1B* region

A

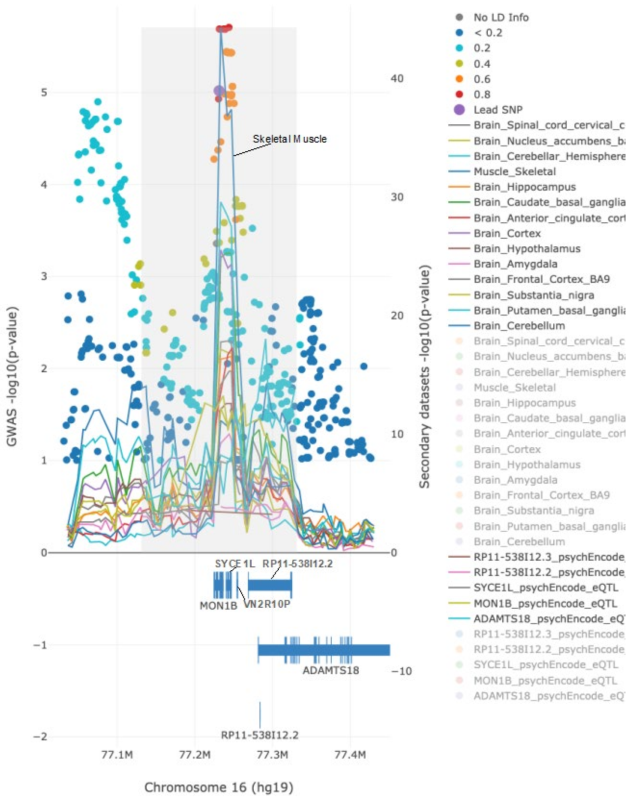

B

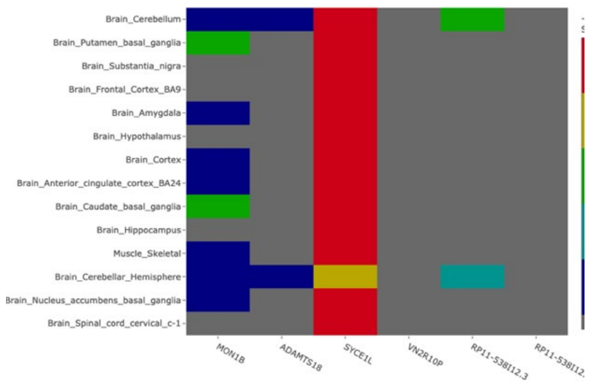

C

Dataset description	Simple Sum -log10P
ADAMTS18_psychEncode_eQTL	3.08e-05
MON1B_psychEncode_eQTL	2.65
RP11-538I12.2_psychEncode_eQTL	1.78
RP11-538I12.3_psychEncode_eQTL	1.02
SYCE1L_psychEncode_eQTL	3.39*

*Colocalization Plots for the *MON1B* region. The highlighted gene for which GTEx eQTLs are displayed in subfigure A is *SYCE1L* and the GWAS results are for MSW. Significant colocalization is determined by the suggested Simple Sum colocalization threshold of 2.78 (-log10 (0.05/#of tests)). PsychENCODE eQTL SS colocalization is significant for the *SYCE1L* gene and borderline significant for the *MON1B* gene, at 2.65. For GTEx tissues significant SS colocalization is seen for *SYCE1L* across all GTEx tissues queried, for RP11-538112.3 in cerrebulum and is borderline significant at 2.77 for *MON1B* in brain_caudate_basal_ganglia and brain_putamen_basal_ganglia.

Supplementary Figure 4. Clustering of Significant Variants (P < 0.01) among Known Speech Genes across CFSRS Tests

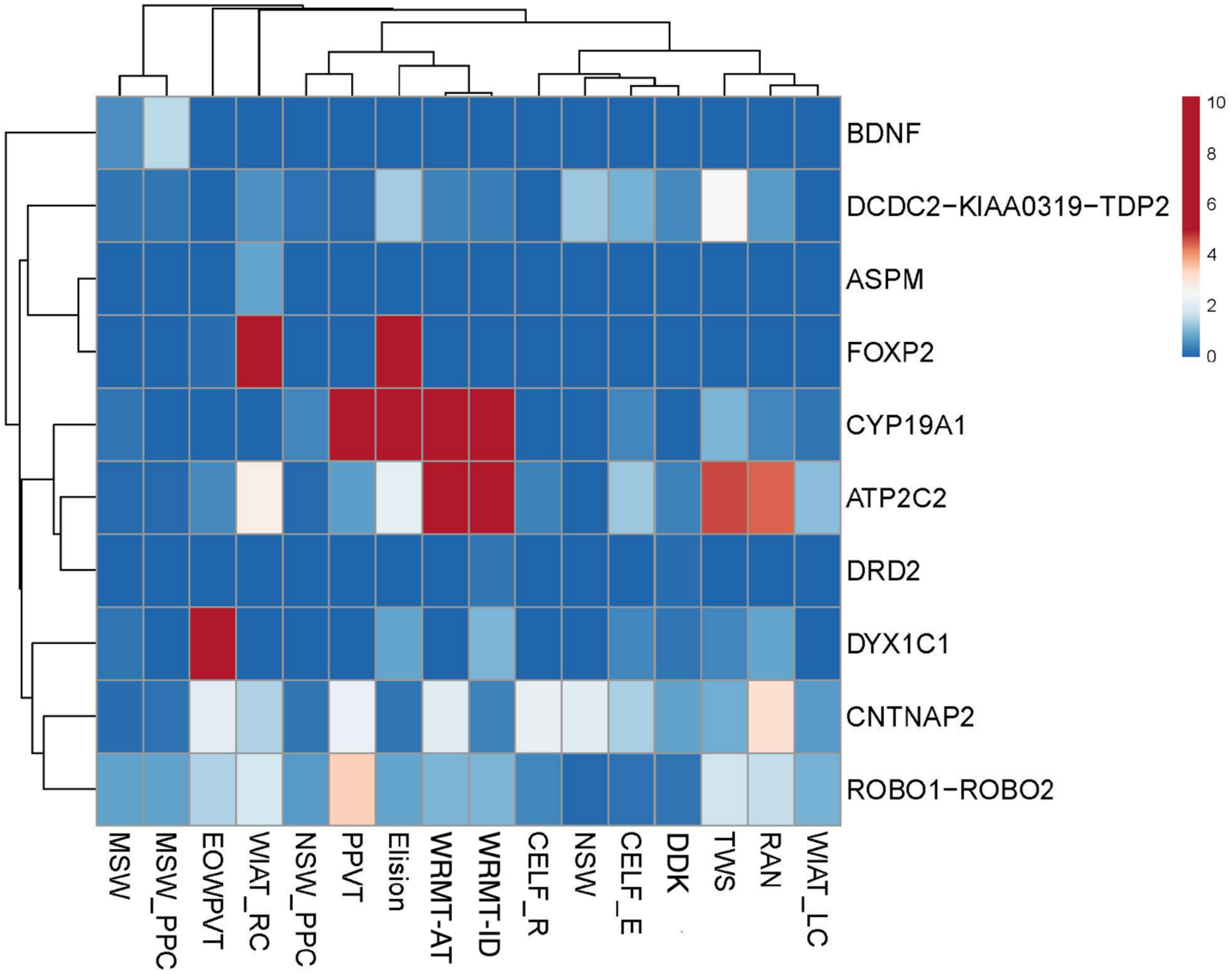

Supplementary Figure 5 – in separate pdf

LocusZoom plots of candidate genes where at least one trait had a SNP significant at $p < 10^{-4}$

Supplementary Figure 6. Clustering of Significant Variants (P < 0.01) among Known Speech Genes across ALSPAC Tests

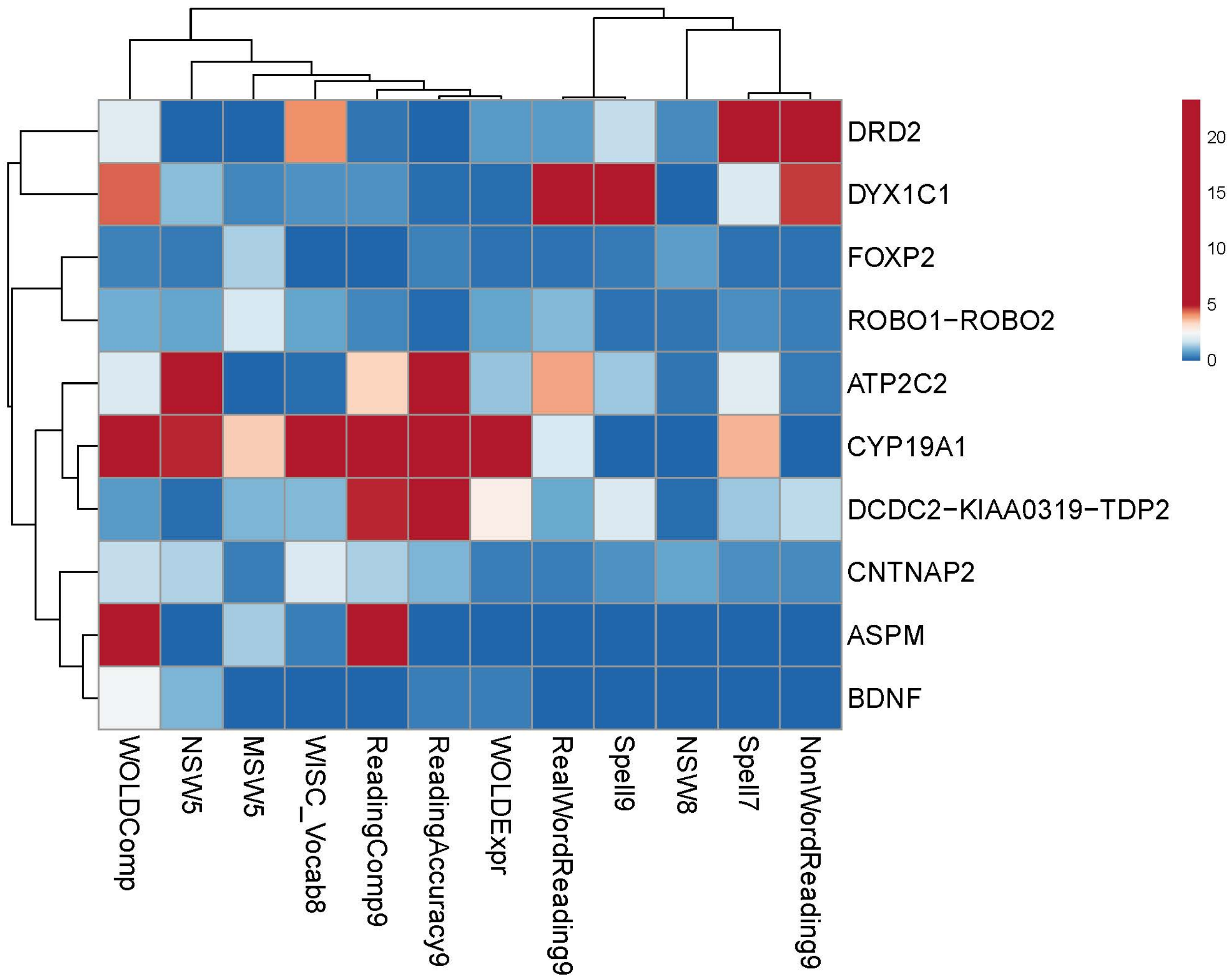

Supplementary Figure 7. Polygenic Risk score across all individual measures

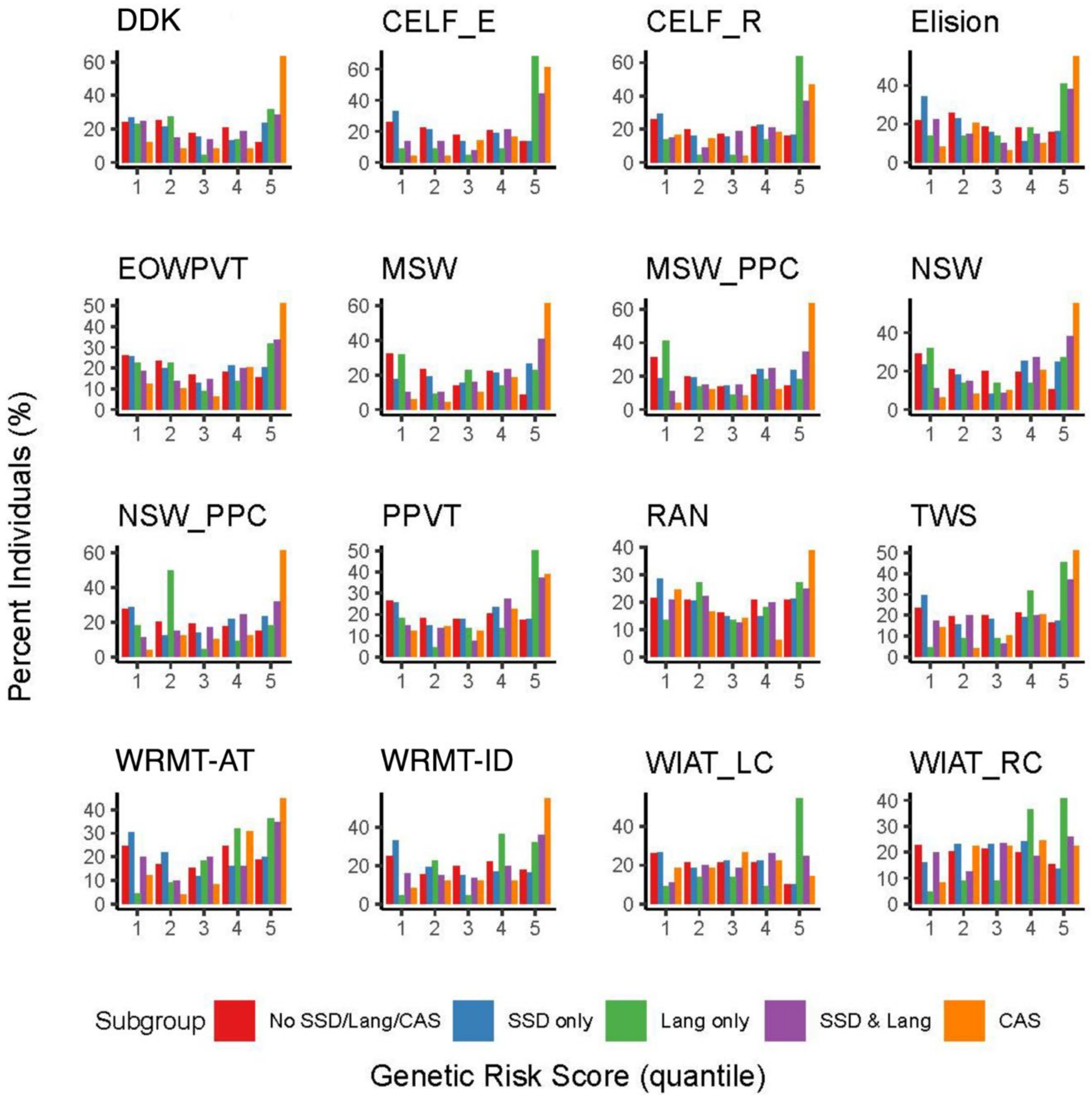

Supplement: Supplementary file 1 — Supplementary Information [file 41525_2021_225_MOESM1_ESM.pdf]
